# Explainable machine learning for incipient anomaly detection in compact molten salt heat exchanger with overlapping feature distributions

**DOI:** 10.1038/s41598-025-27112-8

**Published:** 2026-03-06

**Authors:** Konstantinos Prantikos, Taeseung Lee, Thanh Q. Hua, Lefteri H. Tsoukalas, Alexander Heifetz

**Affiliations:** 1 Nuclear Science and Engineering Division, Argonne National Laboratory, Lemont, IL 60439 USA; 2 School of Nuclear Engineering, Purdue University, West Lafayette, IN 47907 USA

**Keywords:** Engineering, Mathematics and computing

## Abstract

High-temperature molten salt-cooled reactors (MSCRs) are a promising next-generation nuclear technology option, offering efficient power conversion and inherent safety features. However, the reliability of these systems depends on the robust operation of heat exchangers (HXs), which are susceptible to failure due to temperature gradients and channel plugging caused by fluid freezing. Conventional monitoring methods, relying on inlet and outlet measurements, lack the spatial resolution needed to detect early-stage faults. We propose a novel design of a compact salt-to-salt matrix-type HX design consisting of interleaved arrays of parallel tubes, with integrated synthetic fiber optic distributed temperature sensing (DTS) to enable localized detection of incipient faults. To evaluate performance of this design, we generate high-fidelity synthetic data using heat transfer computational modeling to simulate channel plugging, and introduce sensor noise for realistic modeling of measurements. The dataset comprises of 97% normal operation and 3% anomaly cases, with each anomaly class representing 1% of the data. These early anomalies result in overlapping temperature profiles between normal and faulty channels, producing a non-separable dataset that challenges traditional classification techniques. We benchmark eight supervised machine learning (ML) models and demonstrate that XGBoost achieves the highest performance. To improve transparency, we develop an explainability framework combining Shapley values and partially ordered sets (POSETs) to quantify and structurally analyze feature importance. This approach identifies both dominant predictors and ambiguous feature relationships, enhancing trust and interpretability. Our results highlight the potential of combining DTS and explainable ML with intelligent feature selection to improve predictive maintenance and ensure operational resilience in advanced nuclear systems.

## Introduction

High-temperature molten salt-cooled reactors (MSCRs) are promising nuclear energy options for replacement of the aging fleet of commercial light water reactors (LWRs)^1,2^. MSCRs utilize liquid salt process fluids with high melting points (typically above 450 °C) at ambient pressure. Potential advantages of MSCR include more efficient power conversion, compared to the LWR, because of the higher maximum temperature of the thermodynamic cycle (typically 600 °C for MSCR and 275 °C for LWR)^3,4^. In addition, MSCR operation at ambient pressure relaxes requirements on piping and vessel thickness, as compared to those for pressurized LWR (typically 15 MPa pressure). Next to the reactor core, the primary loop heat exchanger (HX) is an essential component for MSCR power plant construction. Nuclear reactor is typically operated in a steady state to minimize stresses on the components. Compared to other components, HX is at a relatively high risk of failure because of the temperature gradients even during steady state operation. If not mitigated through timely repair and maintenance, HX faults can force reactor shutdown. While MSCRs are in the development stage, it is important to design intelligent structural health monitoring (SHM) strategy to reduce future HX operating and maintenance (O&M) costs and ensure MSCR economic viability^5–7^. Combined with advanced sensing, artificial intelligence (AI) and machine learning (ML) have emerged as powerful tools for predictive maintenance and anomaly detection in nuclear systems, offering a new paradigm for improving operational resilience^8–10^. More broadly, advances in temperature sensing technologies have played a central role in enabling diagnostics, monitoring, and control in harsh nuclear environments^11–13^. However, few recent studies have examined MSCR HX options^7,14–18^. A particular unique challenge to the operation of high temperature molten salt HX, which does not affect HX’s of commercial LWRs, is molten salt channel plugging due to fluid freezing, which could result from accumulation of impurities or improper insulation^7,17,19^. Timely detection of incipient plugging faults enables reactor operators to make informed decisions to implement maintenance and remediation strategies.

Conventional HX monitoring involves measuring temperature, pressure and flow rate at the HX inlets and outlets. However, early signs of plugging faults do not lead to measurable changes in the total flow, temperature and pressure. We propose a compact primary loop salt/salt matrix-type HX with embedded distributed temperature sensing (DTS) instrumentation for resilient operation^7,20,21^. The matrix HX consists of interleaved array of parallel tubes, separated by divider plates. The linearity and relative simplicity of the matrix HX geometry allows for installation of distributed temperature sensors to detect and localize faults in the individual channels. The conceptual study in this work is based on high-fidelity synthetic data. Performance of matrix HX is investigated using data generated with COMSOL software. Fault cases of channel plugging are simulated by reducing flow rate in select channels. Temperature values are sampled at a set of points on the divider plate to represent measurements. In an experimental scenario, such measurements could be performed with distributed fiber optic sensor (DFOS)^22,23^. Advantages of DFOS over conventional thermocouple arrays include higher spatial sampling density and fewer physical penetrations through thermal hydraulic components pressure boundaries^24,25^. To develop a realistic representation of sensor measurement data, noise is added into the simulated data^26^.

The challenge in detection and classification of incipient or early-stage faults, which are assumed to involve partially plugged isolated channels, consists of subtle differences temperature values between normal and plugged channels due to diffusion of heat. Detection of anomaly can be accomplished with unsupervised machine learning (ML), where ML trained on normal data identifies outliers during monitoring^27,28^. However, their utility diminishes in early detection scenarios involving subtle anomalies, especially when feature distributions are non-separable and exhibit substantial overlap between normal and anomalous cases. Such overlapping distributions pose significant challenges, particularly when anomalies lack distinct, easily identifiable characteristics, which is the focal point of this investigation. In addition, while unsupervised methods may signal the presence of an anomaly, they do not provide information to the reactor operator about the degree of channel plugging. Informing the operator about the level of damage can be achieved through classification with supervised learning. In this work, we explore the feasibility of anomaly detection and classification using supervised learning. Recent advances in supervised ML have achieved state-of-the-art performance on challenging forecasting and classification problems^29–31^. We benchmark eight supervised ML models, which include Logistic Regression (LR), K-Nearest Neighbors (KNN), Gaussian Naïve Bayes (GNB), Support Vector Machine (SVM), Decision Trees (DT), Random Forest (RF), Feed-Forward Neural Network (FNN), and Extreme Gradient Boosting (XGBoost). Our results demonstrate that the XGBoost classifier achieves the best performance. Classification performance efficiency increases for HX channels with higher degree of plugging.

To improve model transparency and support operator decision-making, we introduce an explainability framework that combines Shapley values with Partially Ordered Sets (POSETs). Shapley values, rooted in cooperative game theory, quantify the contribution of each input feature to individual predictions by averaging marginal contributions across all possible feature combinations^32,33^. While they provide a robust measure of local and global importance, Shapley values impose implicitly a total ranking of features, even when differences are marginal or statistically insignificant^34,35^. In safety-critical applications such as nuclear heat exchanger monitoring, this can lead to overconfident interpretations of model behavior, where small, possibly noisy differences are mistakenly treated as meaningful. To mitigate this risk and to capture ambiguity in feature importance, we incorporate a complementary analysis using POSETs. POSETs provide a formal mathematical framework for representing partial rankings, allowing us to explicitly model feature dominance, equivalence, and incomparability^36,37^. In this study, we construct class-specific POSETs based on SHAP values and visualize them using Hasse diagrams to reveal hierarchical structures and clusters of features with overlapping predictive influence. This hybrid approach enables class-aware interpretability and clearly separates confidently ranked features from ambiguous ones, an essential capability in safety-critical applications such as nuclear heat exchanger monitoring. By combining Shapley values with POSETs, our framework not only identifies which features drive predictions but also highlights uncertainty in their relative influence, thereby improving trust and decision-making support for reactor operators.

This paper is structured as follows. The “Results” section presents the classification results and summarizes performance benchmarking of all classifiers. The “Discussion” section introduces the HX plugging fault cases, offers insights into the dataset details, and interprets model predictions through the proposed explainability framework using Shapley values and POSETs. The “Methods” section describes the ML models, classification metrics, molten salt HX model, sensor noise analysis, and implementation of the explainability tools deployed in this study.

## Results

A total of 36 different steady state instances of the molten salt HX were generated using COMSOL heat transfer and pipe modules, in which we added corresponding noise to each sensor. The testing dataset has a 5.24% ratio of minority-to-majority class, comprising 4998 instances and encompassing nine distinct features. The testing dataset consisting of 4749 instances of open channels (class 0) and 249 instances of plugged channels (class 1, class 2, and class 3) was excluded from the training phase. Plugged channels are further categorized based on the extent of plugging: 20% plugged flow rate with 82 instances (class 1), 40% plugged flow rate with 84 instances (class 2), and 60% plugged flow rate with 83 instances (class 3). A significant challenge encountered in ML with imbalanced class distributions is the inherent bias of most conventional algorithms towards optimizing for accuracy. This predisposition leads to a focus on minimizing overall error rates with the aim of enhancing classification accuracy. However, in scenarios characterized by an imbalanced class distribution, relying solely on classification accuracy as a metric can yield deceptive results. The exclusive emphasis on accuracy in such contexts may not only provide an incomplete picture of a classifier’s effectiveness but also propagate misleading interpretations of its performance. To address this issue and ensure a more comprehensive assessment, our approach incorporates the use of confusion matrices, F1-scores, precision-recall (PR) curves, and area under the precision-recall curve (AUC-PR) metrics, offering a multidimensional perspective on classifier performance.

**Fig. 1:**
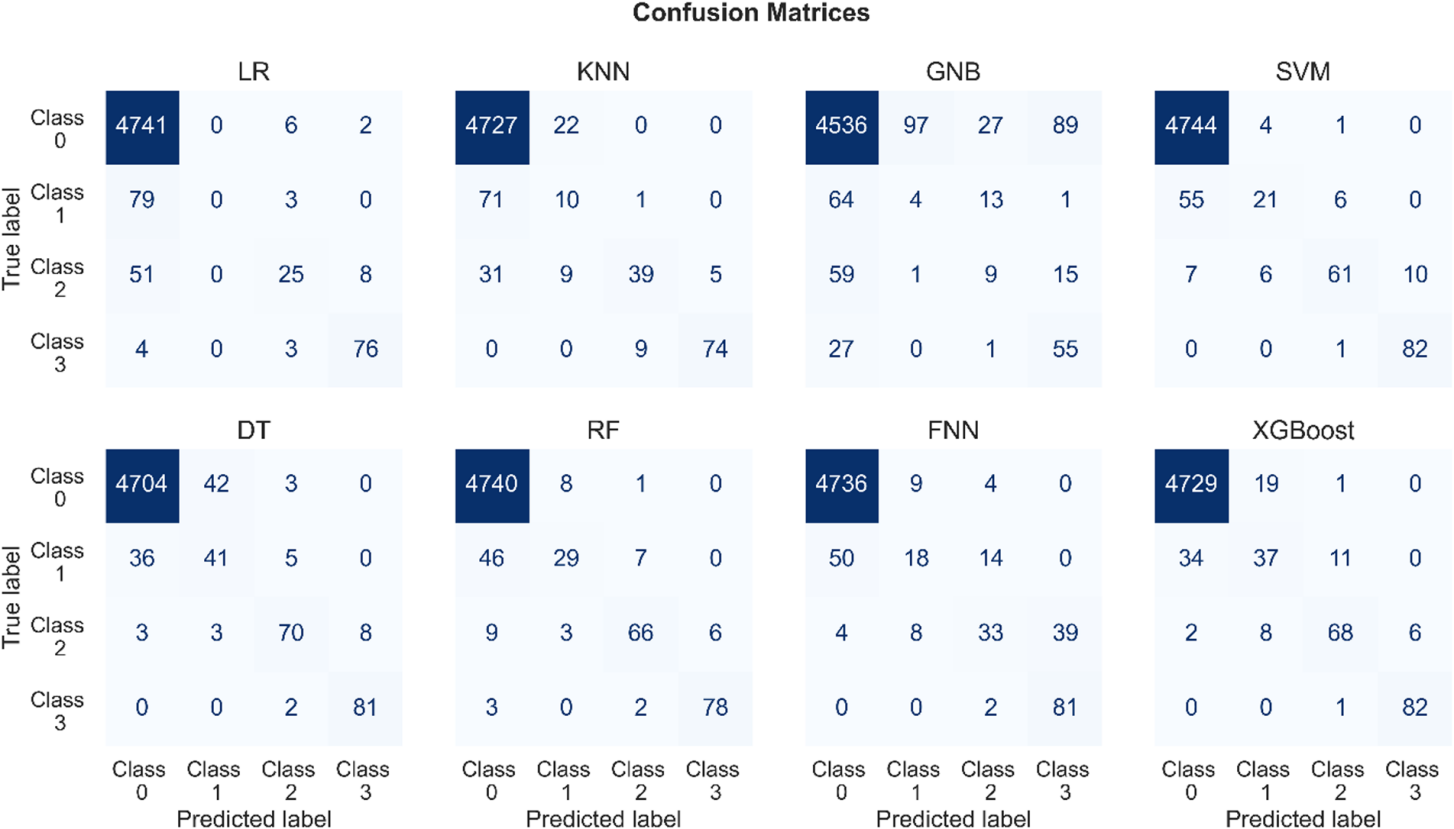
Confusion matrices for eight ML models, showing the number of correct and incorrect predictions for each of the four classes. Misclassifications are concentrated primarily in early-stage fault classes, reflecting the inherent difficulty in distinguishing subtle anomalies with overlapping feature distributions.

Figure 1 presents the confusion matrices generated using predictions from eight different ML algorithms on the testing dataset. For multi-class classification, each confusion matrix captures the performance of the models across all classes, with rows representing the true class labels and columns indicating the predicted class labels. The diagonal elements of the matrix correspond to the true positive (TP) outcomes for each class, reflecting correctly classified instances. Off-diagonal elements represent misclassifications, with false positives (FP) and false negatives (FN) distributed across non-diagonal entries. The total number of FP for a specific class can be calculated by summing the elements in the column corresponding to that class, excluding the diagonal element. For example, for class 1, calculating the FP represents instances where other classes were incorrectly predicted as class 1. Similarly, the total number of FN for a specific class is the sum of the elements in the row corresponding to that class, excluding the diagonal element. For instance, for class 1, calculating the FN represent instances of class 1 that were misclassified into other classes.

Table 1 presents a comparison of FP and FN errors across all four classes for different algorithms. In the context of nuclear safety, misclassifying a plugging instance as normal operation (FP for class 0) is the most critical error, as it directly impacts the ability to identify and mitigate plugging events. Therefore, minimizing FP for class 0 is the highest priority. Among the evaluated algorithms, XGBoost achieves the lowest FP error for class 0 with just 36 cases, followed by DT, RF, and SVM with FP errors of 39, 58, and 62, respectively. This demonstrates xgboost’s superior ability to correctly classify normal operation instances, reducing false alarms in the majority class. Additionally, it is important to minimize FN for class 0, where normal operation instances are misclassified as plugging. The lowest FN error for class 0 is achieved by SVM with only 5 cases, indicating its strong recall in the majority class. LR, RF, and FNN follow closely with FN errors of 8, 9, and 13, respectively. However, it is worth noting that while SVM performs well in FN for class 0, it exhibits higher FP in this class, showing a tradeoff in performance. For minority classes (1, 2, and 3), minimizing both FP and FN is crucial to ensure accurate detection and localization of plugging faults. XGBoost demonstrates strong performance across these classes. For example, it achieves competitive FP and FN values for class 1 (27 and 45, respectively) and class 2 (13 and 16, respectively), highlighting its balanced performance in handling class imbalance. Class 3 sees some of the lowest FP and FN errors overall, with XGBoost achieving only 6 FP and 1 FN, matching the best FN performance reported by SVM. Overall, XGBoost emerges as the most consistent algorithm, achieving the best results in the majority class (class 0) for FP while maintaining competitive performance in minority classes. Finally, KNN, LR, and GNB classifiers were found to be the weakest performers by a significant margin.

**Table 1:** Comparison of FP and FN errors for each anomaly class (0–3) across eight ML algorithms.

Metric	Algorithm	LR	KNN	GNB	SVM	DT	RF	FNN	XGBoost
	Class								
False Positives	0	134	102	150	62	39	58	54	**36**
	1	**0**	31	98	10	45	11	17	27
	2	12	10	41	**8**	10	10	20	13
	3	10	**5**	105	10	8	6	39	6
False Negatives	0	8	22	213	**5**	45	9	13	20
	1	82	72	78	61	**41**	53	64	45
	2	59	45	75	23	**14**	18	51	16
	3	7	9	28	**1**	2	5	2	**1**

Table 2 summarizes the classification metrics of precision, recall, and F1-score for all four classes across the eight ML classifiers. These metrics provide insight into the classifiers’ ability to handle both the majority class 0 and the minority classes 1, 2, and 3 in the multi-class setting. According to the precision metric, which reflects the accuracy of positive predictions, XGBoost demonstrates strong performance, achieving class 0 (0.99), class 2 (0.84), and class 3 (0.93), while ranking third for class 1 (0.58). RF achieves the highest precision for class 1 (0.72), while DT and SVM also perform well for class 2 and class 3 (both achieving 0.88 or higher). Although the FNN ranks fourth for Class 1 (0.51), it maintains moderate precision across all minority classes. KNN shows good precision for class 3 (0.94), but its performance declines for other classes, particularly class 1. GNB struggles across most classes, with class 1 (0.04) reflecting particularly low precision.

For recall, which measures the ability to capture all true positives, XGBoost achieves perfect recall for class 0 (1.00), as do almost all classifiers. XGBoost and SVM excel with the highest recall of class 3 (0.99). However, recall remains challenging for class 1, with DT performing best for class 1 (0.50), followed by XGBoost for class 1 (0.45). Tree-based algorithms again outperform others, whereas GNB and LR exhibit significant limitations for minority classes, particularly for class 1 and class 2, where recall values drop below 0.30. For the F1-score, which balances precision and recall providing an overall performance metric, all algorithms achieve strong results for class 0 (0.96–0.99). For class 3, XGBoost leads with the highest F1-score for class 3 (0.96), followed by DT (0.94), SVM (0.94), and RF (0.93). For class 2, DT achieves the best F1-score for class 2 (0.85), with RF and XGBoost closely following. Class 1 remains the most challenging, where XGBoost outperforms with class 1 (0.51), but overall F1-scores remain low across all algorithms.

**Fig. 2:**
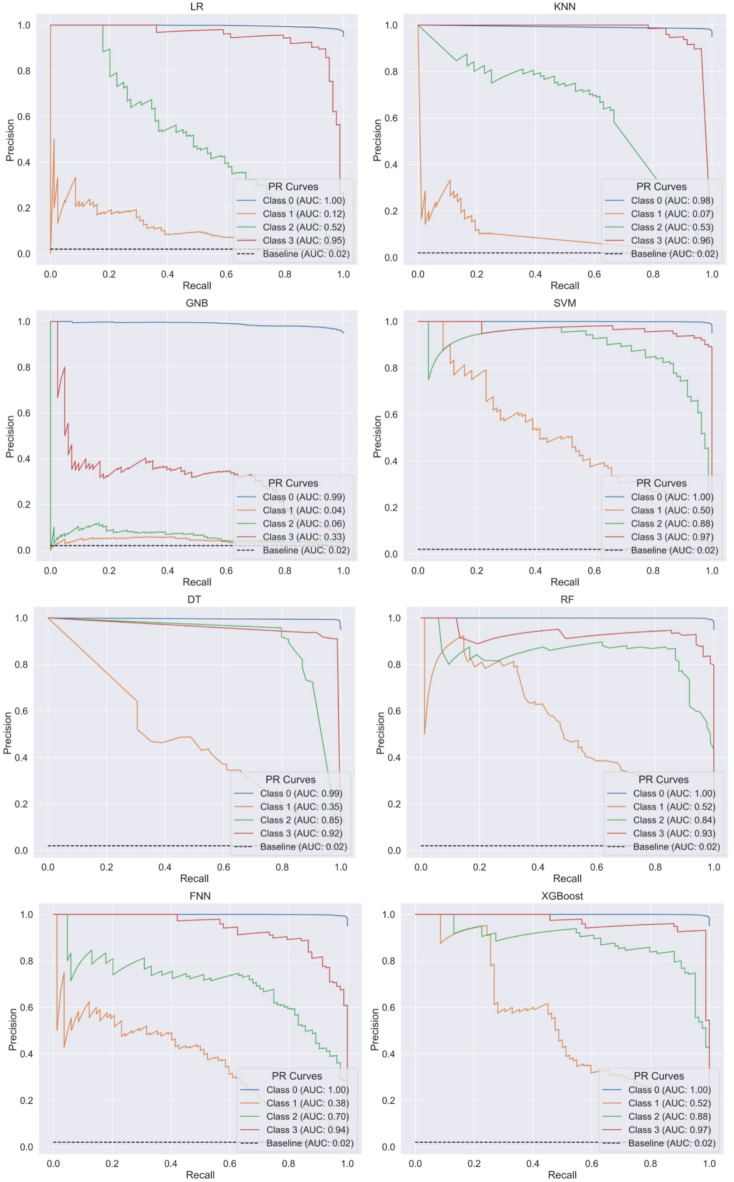
Precision-recall curves and their respective AUC-PR values for eight ML classifiers.

To enhance the quantification of the classification performance, Fig. 2 displays the PR curves and their respective AUC-PR values for different ML algorithms. The steepness associated with PR curves reflects the model’s efficacy in optimizing precision while minimizing recall. A higher AUC-PR value signifies an enhanced capacity to strike a balance between precision and recall. The baseline AUC-PR for each minority class (representing plugged channels) is 0.02, which corresponds to a random or dummy classifier that consistently predicts instances as belonging to one of the minority classes (Classes 1, 2, and 3).

**Table 2:** Per-class performance metrics for eight ML algorithms.

Metric	Algorithm	LR	KNN	GNB	SVM	DT	RF	FNN	XGBoost
	Class								
Precision	0	0.97	0.98	0.97	**0.99**	**0.99**	**0.99**	**0.99**	**0.99**
	1	0.00	0.24	0.04	0.68	0.48	**0.72**	0.51	0.58
	2	0.68	0.80	0.18	**0.88**	**0.88**	0.87	0.62	0.84
	3	0.88	**0.94**	0.34	0.89	0.91	0.93	0.68	0.93
Recall	0	**1.00**	**1.00**	0.96	**1.00**	0.99	**1.00**	**1.00**	**1.00**
	1	0.00	0.12	0.05	0.26	**0.50**	0.35	0.22	0.45
	2	0.30	0.46	0.11	0.73	**0.83**	0.79	0.39	0.81
	3	0.92	0.89	0.66	**0.99**	0.98	0.94	0.98	**0.99**
F1-score	0	**0.99**	**0.99**	0.96	**0.99**	**0.99**	**0.99**	**0.99**	**0.99**
	1	0.00	0.16	0.04	0.37	0.49	0.48	0.31	**0.51**
	2	0.41	0.59	0.13	0.80	**0.85**	0.82	0.48	0.82
	3	0.90	0.91	0.45	0.94	0.94	0.93	0.80	**0.96**

Bolded values denote the highest performing classifier for each metric and class.

The XGBoost classifier demonstrated the highest overall performance in this multi-class classification task, achieving AUC-PR values for class 0 (1.00), class 1 (0.52), class 2 (0.88), and class 3 (0.97). RF closely followed, with AUC-PR scores for class 0 (1.00), class 1 (0.52), class 2 (0.84), and class 3 (0.93). SVM delivered competitive results, achieving class 2 (0.88) and class 3 (0.97), while DT performed well with class 2 (0.85) and class 3 (0.92). FNN showed moderate performance for class 2 (0.70) and strong performance for class 3 (0.94). These results highlight the strong performance of tree-based classifiers (XGBoost, RF, and DT) and SVM in distinguishing both majority and minority classes. Notably, all models consistently achieved near-perfect AUC-PR values for class 0, underscoring their ability to handle the majority class effectively.

In contrast, all classifiers struggled significantly with the minority class 1, which consistently exhibited the lowest AUC-PR values. XGBoost and RF demonstrated the best performance for the most challenging class, both achieving class 1 (0.52), with SVM following closely with class 1 (0.50). Other models, such as FNN with class 1 (0.38) and DT with class 1 (0.35), showed limited ability to accurately identify instances from this minority class. Simpler classifiers, including GNB and KNN, performed poorly for both class 1 and class 2, with GNB achieving class 1 (0.04) and class 2 (0.06), and KNN scoring class 1 (0.07) and class 2 (0.53). LR, while effective for the minority class 3 (0.95), struggled with class 1 (0.12) and class 2 (0.52). These results underscore the difficulty of accurately classifying underrepresented classes, particularly when using simpler models. Despite the comparatively low AUC-PR values for the minority class 1, even the underperforming classifiers exceeded the baseline AUC-PR score of 0.02, demonstrating that their performance, while moderate, was still meaningful in addressing the classification task.

**Fig. 3:**
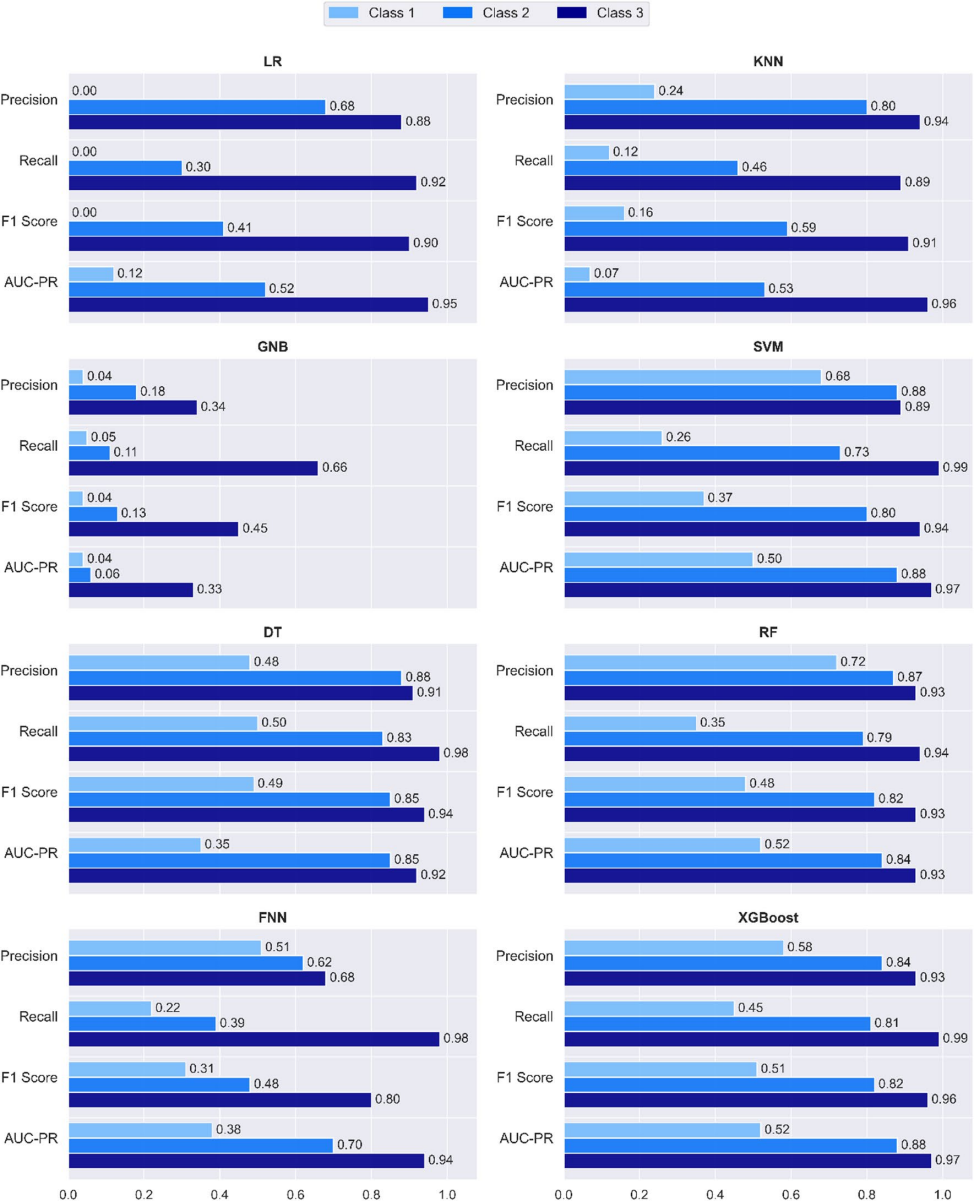
Summary of metric values across minority anomaly classes (class 1, class 2, and class 3).

Figure 3 summarizes the classification metrics of precision, recall, F1-score, and AUC-PR for the minority classes (class 1, class 2, and class 3) across the eight ML classifiers. Class 0 is excluded, as most classifiers achieve near-perfect scores for this class across all metrics.

When evaluating precision, recall, F1-scores, and AUC-PR values collectively, XGBoost clearly emerges as the most effective classifier, exhibiting a robust ability to handle imbalanced and skewed classes. Following closely in performance are the tree-based classifiers RF and DT, which also delivered strong results across multiple classes. SVM and FNN, while not on par with XGBoost, demonstrated notable performance, particularly for class 3. These findings underscore the advantage of advanced ensemble-based and tree-based methods in tackling the inherent challenges of multi-class classification in imbalanced datasets with overlapping distributions.

In addition, classification algorithms considered in our paper can perform in real time to detect rapid freezing events that occur within minutes. The most accurate classification algorithm in our study, XGBoost, performs inference in non-iterative and scales with the number and depth of trees. For our configuration (200 trees, max depth = 5), a single prediction involves in the order of 10^3^ node comparisons, which is sub-millisecond on a standard CPU. In our micro-benchmarks, end-to-end latency per steady-state snapshot (feature aggregation from DFOS + model scoring) is well below typical SCADA update intervals (seconds), and the memory footprint is modest.

## Discussion

**Fig. 4:**
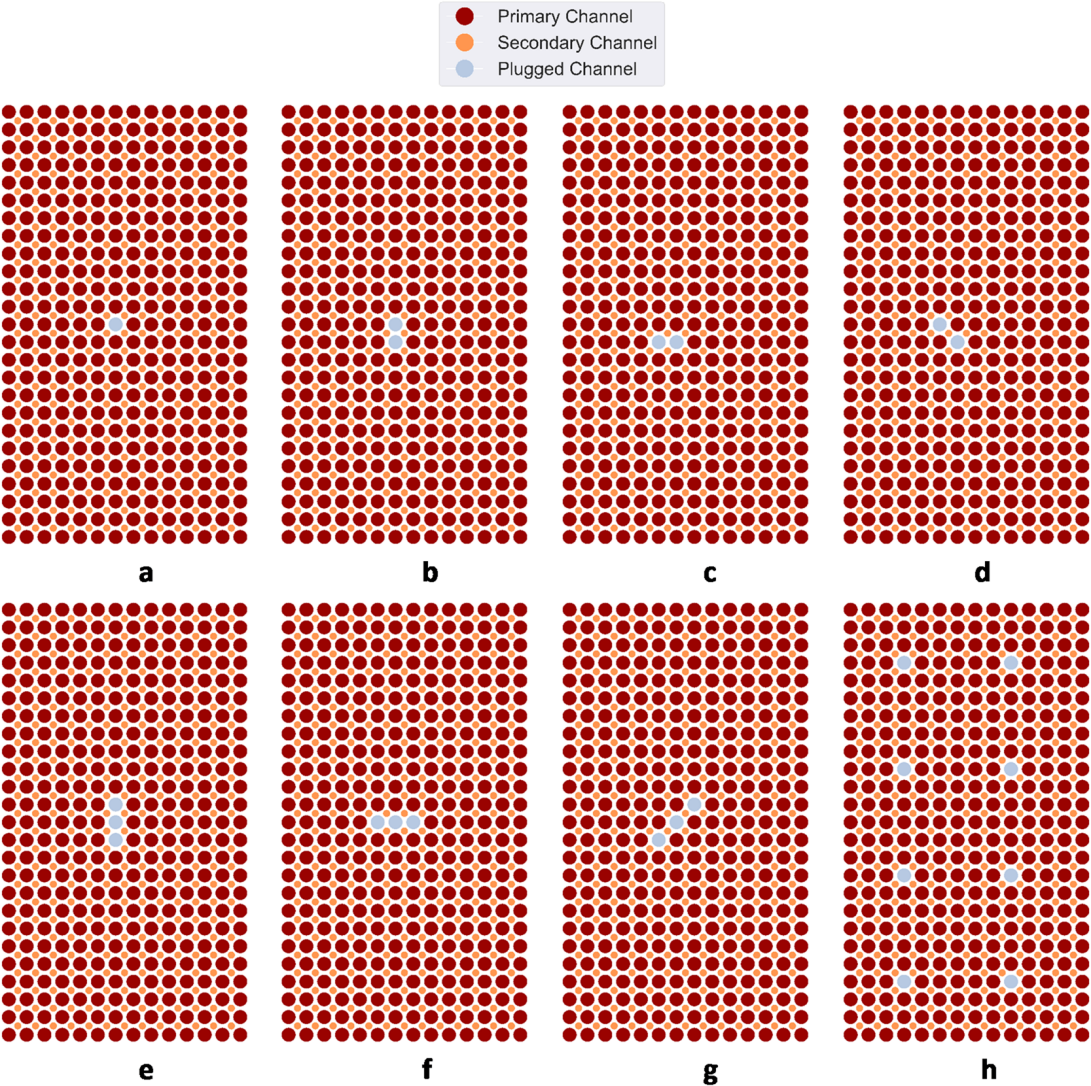
Schematic visualization of plugging fault cases considered in this study. The grey color indicates a plugged channel, while the red color and orange color represents fully open primary and secondary channels respectively. Cases (**a**)–(**h**) were used in operational scenarios with a single plugging flow rate at a time, considering flow rates of 40%, 60%, and 80%. In total 24 fault cases were created out of these configurations.

**Fig. 5:**
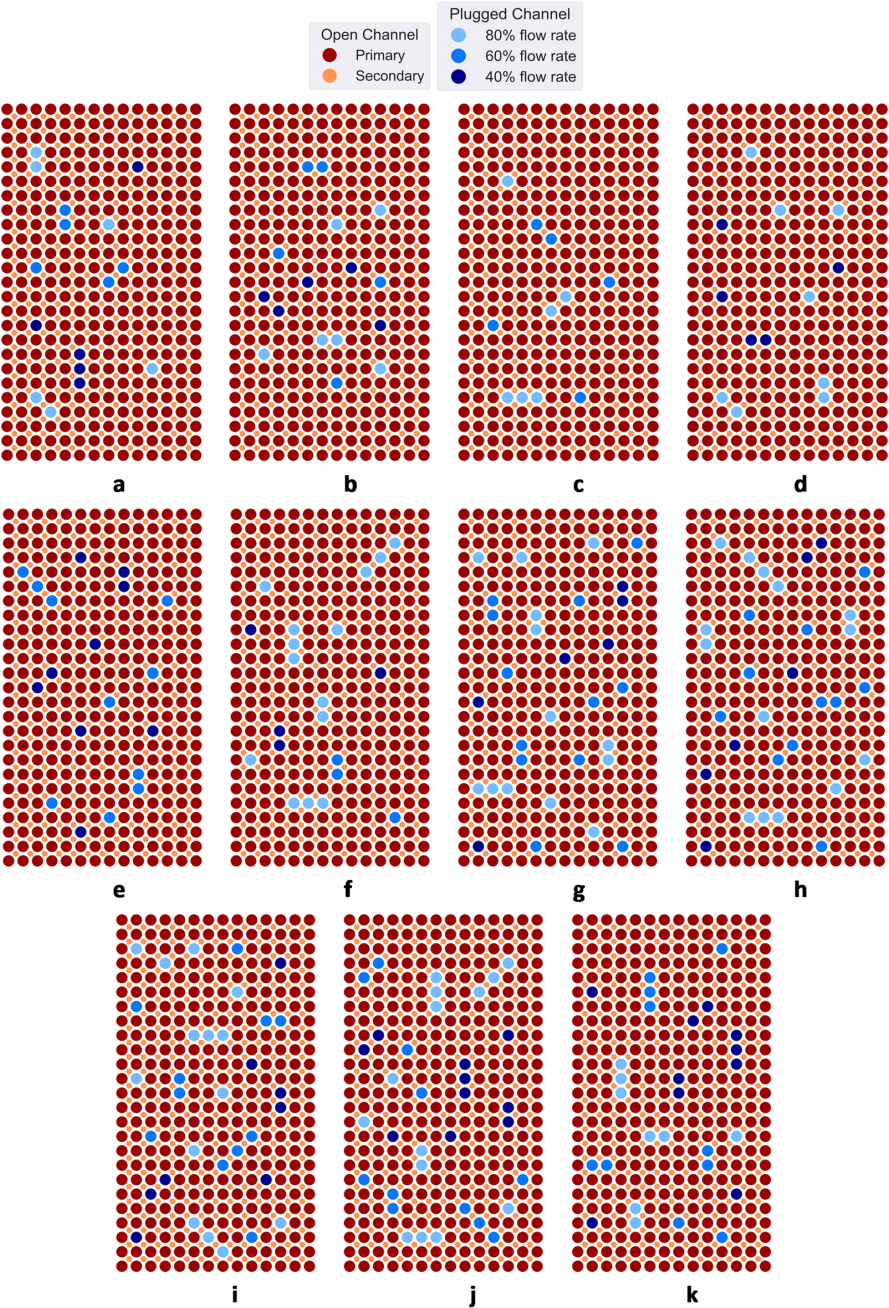
Schematic visualization of plugging fault cases considered in this study. The blue color indicates a plugged tube, while the red color represents a fully open tube. Cases (**a**)–(**k**) were used in operational scenarios with multiple plugging flow rates at a time, resulting from combinations of the three distinct flow rates.

In this paper, we have demonstrated that a novel design of molten salt HX with embedded DTS instrumentation, intelligent feature selection, and explainable ML that uses Shapley values with POSETs can achieve automation of incipient anomaly detection in a challenging anomaly detection task. A total set of 36 channel plugging fault cases was constructed for this study. We incorporated three different categories of channel flow reduction, involving flow rates of 40%, 60%, and 80%. We selectively performed plugging of primary channels in several locations of the HX. The architecture of the matrix heat exchanger consists of interleaved array of parallel channels. Because of the repetitive structure, we assume that a plugging fault is a stochastic event that can occur on any of the channels with an equal probability. The approach in our study consists of physics-agnostic detection and classification of channel plugging faults that may occur in any a-priori unknown channels/regions. Multiple variations of plugging faults that resulted in partial blockage of the primary channels were considered in this study. Figures 4 and 5 shows representative plugging fault cases examined in this study. Within the scenarios presented in Fig. 4a–h, totaling 8 cases, we conducted separate operational assessments using a single plugging flow rate at a time. These evaluations encompassed flow rates of 40%, 60%, and 80%, thus yielding a total of 24 cases. In cases depicted in Fig. 5a–k, we considered unique operational scenarios featuring variable plugging flow rates, resulting from combinations of the three distinct flow rates. In Fig. 4, the grey color indicates a plugged primary channel, while the red color represents a fully open primary channel and the orange color represents a fully open secondary channel. In Fig. 5, the different nuances of blue indicate different plugging flow rates. The darkest nuance signifies greater plugging flow rate.

The dataset developed with COMSOL simulations has nine predictor variables and one target variable. We initially evaluated 14 predictor variables and retained nine after ablation for stability/performance under overlapping distributions. Excluded variables were HX primary inlet temperature measured with thermocouple (TC), HX secondary inlet temperature measured with TC, HX primary-side pressure drop measured with pressure gauge, Primary loop flow rate, and Secondary loop flow rate. Table 3 lists the retained variables and their definitions. The first four predictor variables listed in the table are collectively referred to as contextual features. These features provide the context about the conditions and setup under which the data was collected, rather than measurements of fluid thermophysical properties such as temperature or pressure. The gauge location and gauge proximity describe the spatial configuration of the sensors relative to the heat exchanger system topology, providing insight to where sensors should be installed in the experimental system for optimal measurements. The measurement number acts as a separator for different fault scenarios within the dataset. Note that there is no temporal correlation between measurements, and their order can be changed. The divider plate number together with the gauge location serve as the coordinate variables for localization of the channel plugging fault.

**Table 3:** Description of dataset features used for multi-class classification.

Type	Variable	Description
Predictor	Gauge location	Gauge position along DFOS [cm]
	Gauge proximity	Binary indicator of proximity to either primary or secondary channel [unitless]
	Measurement number	Measurement number [unitless]
	Divider plate number	HX divider plate number [unitless]
	Primary inlet distributed temperature	Primary hot side inlet manifold (secondary cold side outlet manifold) temperature measured across divider plates with DFOS [°C]
	Primary outlet distributed Temperature	Primary cold side outlet manifold (secondary hot side inlet manifold) temperature measured across divider plates with DFOS [°C]
	HX primary outlet temperature	Temperature of HX primary combined outlet measured with thermocouple [°C]
	HX secondary outlet temperature	Temperature of HX secondary combined outlet measured with thermocouple [°C]
	HX secondary outlet pressure drop	Pressure drop across secondary system measured with pressure transmitter [Pa]
Target	Plugging classification	Open channels (class 0) or plugged channels (classes 1, 2, and 3)

COMSOL computer simulations produced temperature and pressure distribution for each fault scenario. The temperature readings from 1428 gauges, located at the front and back of the HX tubing manifold. In principle, DFOS allows for much higher spatial density of sampling points. However, incorporating readings from all gauges would result in high rate of false alarms. The reason is that partial plugging of the channels leads to local divider plate temperature change on the order of several degrees, just above the uncertainty level for a DFOS temperature sensing. The change in temperature is largest at the locations on the divider plate closest to the contact points with the plugged channel. The target variable marks the channels as open (class 0) or plugged (class 1, 2, and 3) depending on the grade of partial plugging.

**Fig. 6:**
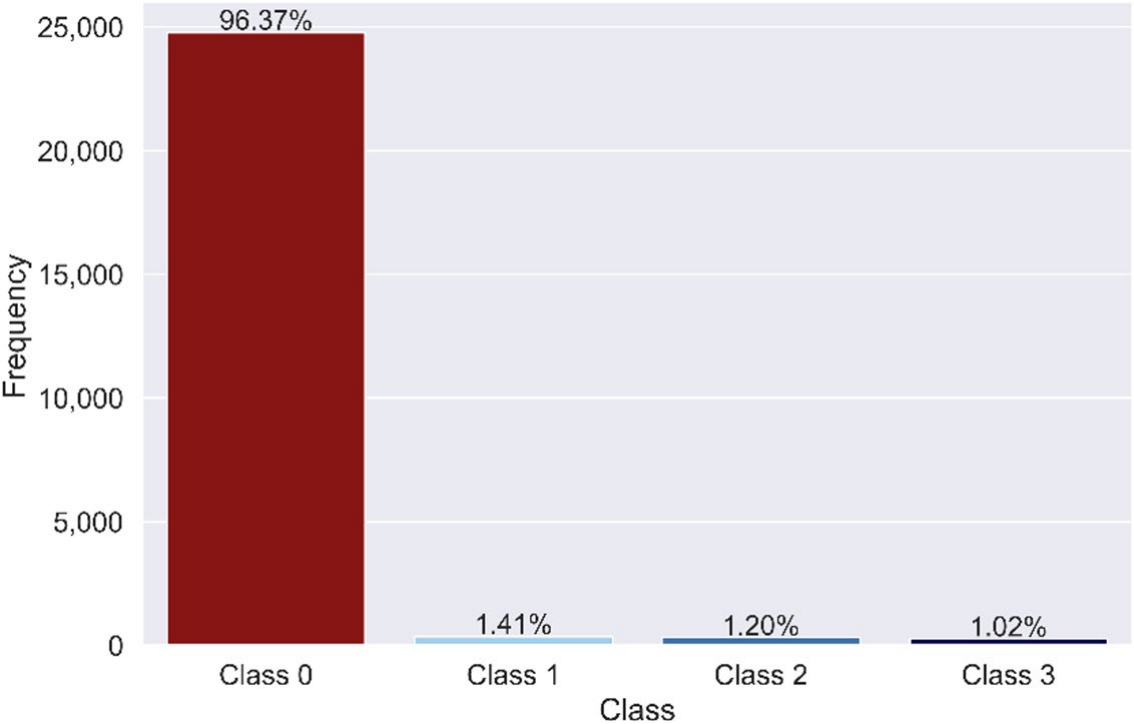
Class distribution of the plugging dataset. Histogram illustrating the severe class imbalance present in the dataset. This pronounced skew underscores the importance of designing classifiers that are robust to rare event detection and minimizing bias toward the majority class.

In Fig. 6, we present the categorical observations from our dataset, involving all 36 cases. One of these cases represents the scenario of normal operation without any instances of channel plugging. It is evident that the instances of open channels significantly outnumber those of plugged channels. Specifically, the dataset comprises 25,704 instances, with 24,772 classified as open channels, in stark contrast to the 932 instances identified as plugged channels, resulting in a minority-to-majority class ratio of 3.76%. This corresponds to class distributions of 1.41% for class 1, 1.20% for class 2, and 1.02% for class 3. This substantial imbalance in our dataset highlights the considerable challenge associated with effectively detecting plugged channels. Nonetheless, it is important to note that in real-world operational scenarios, a multitude of plugged tubes would not be a common occurrence simultaneously. If such a situation were to arise, the resulting drop in operational efficiency would serve as a clear indicator of an uncertain condition within the heat exchanger. Therefore, we structured our dataset to closely resemble real-world operational scenarios, recognizing the rarity of simultaneous occurrences of plugged tubes in practical applications.

**Fig. 7:**
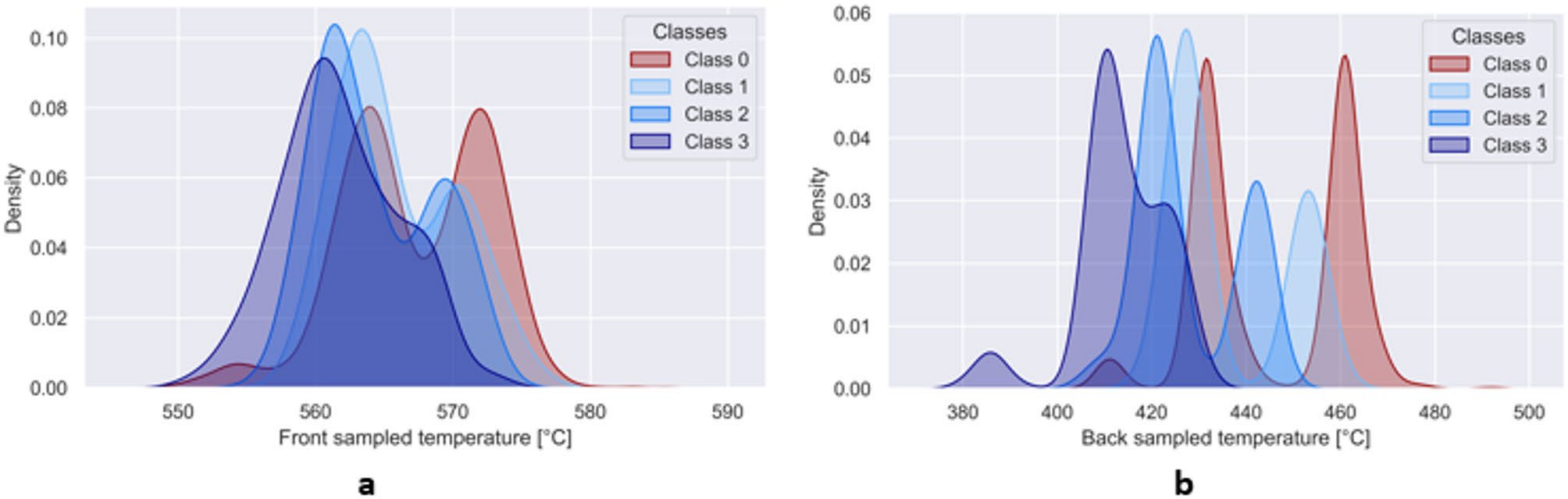
(**a**) Density distributions of front sampled temperature for all classes. (**b**) Density distributions of back sampled temperature for all classes.

To model the experimental measurement scenario with higher fidelity, in this work we introduce sensor noise into COMSOL-simulated HX data^26^. This involves injecting brown noise into all DTS readings associated with fiber optic sensors, injecting pink noise into the temperature readings associated with thermocouples, and white noise into the pressure readings associated with pressure gauges. The primary impact of plugged channels manifests in temperature alterations. Therefore, in Fig. 7a and b we present the kernel density estimate (KDE) plots illustrating the relationship between front sampled temperature and plugging distribution, as well as back sampled temperature and plugging distribution, respectively. The distributions shown in both figures demonstrate that there is no clear threshold where the differentiation between open and plugged channels becomes evident. The overlapping occurrence of open and plugged channel cases further underscores the significant challenge associated with detecting plugged channels.

**Fig. 8:**
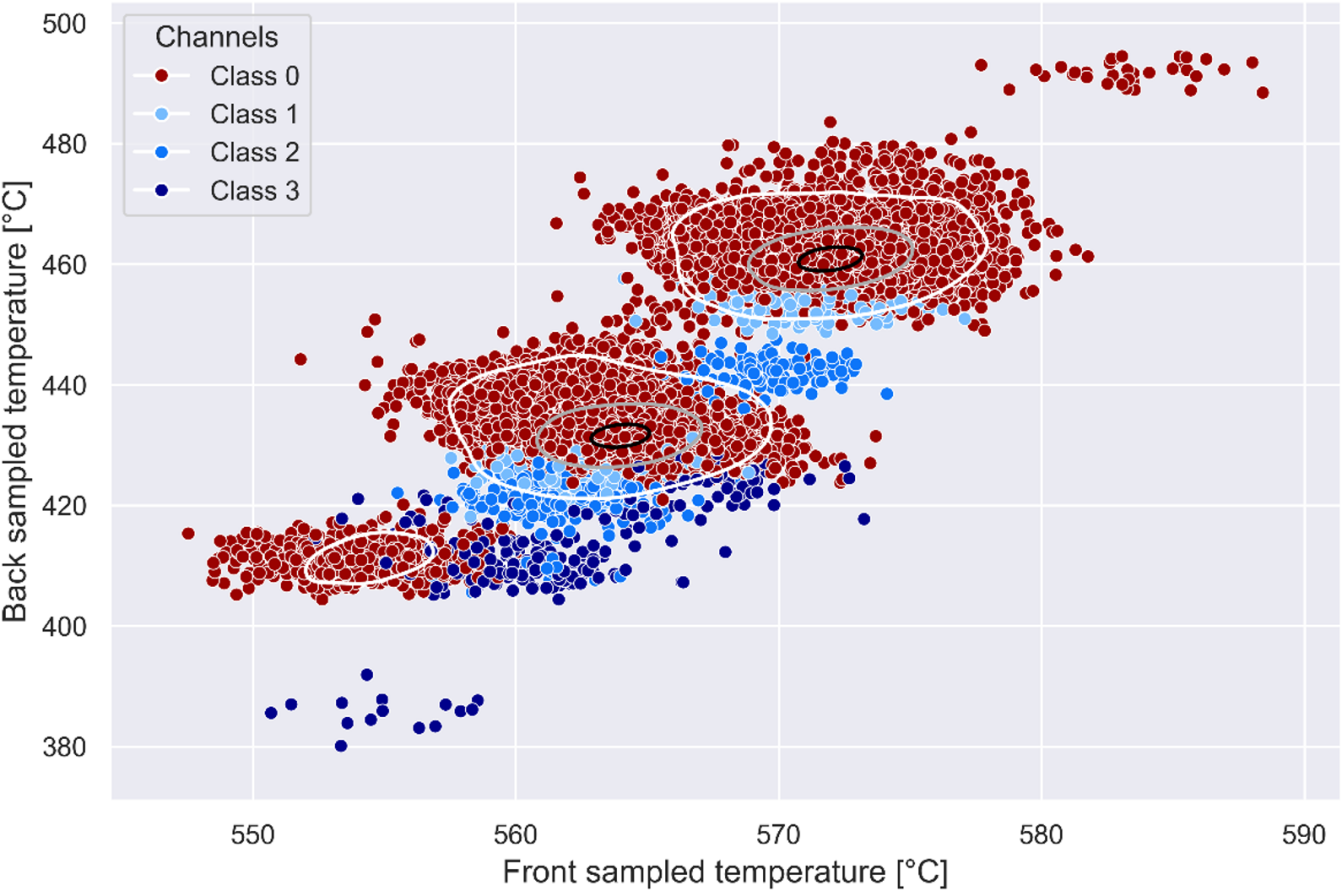
Dataset visualization in temperature feature space (open vs. plugged channels).

To illustrate the complexity of the current dataset, Fig. 8 provides a representation of the distribution of open and plugged channels. The contour lines provide insights on the dataset distribution. Specifically, the two black contour lines include inside them the 10% of the dataset, the two grey contour lines include inside them the 70% of the dataset, and the three white contour lines include inside them the 90% of the dataset. There are multiple instances where the temperature distributions of open and plugged channels overlap, exhibiting almost identical temperature. Several factors contribute to this overlapping effect.

First, cases involving channel plugging at an 80% flow rate (class 1) are particularly sensitive. This sensitivity arises from the minimal temperature change when compared to channels with a 100% flow rate, i.e., fully open channels. Considering the injection of brown noise, even the slightest temperature variation can become obscured, making the detection of channel plugging at 80% flow rate a challenging task. The intricacy of the task escalates further in instances where detection is carried out for singular channel plugging without concurrent plugging in nearby channels.

Second, in cases involving channel plugging at 60% flow rate (class 2) and 40% flow rate (class 3), adjacent channels situated in close proximity, especially within the divider, are affected by the plugging and consequently exhibit temperature differences compared to channels with a 100% flow rate. The extent of this temperature difference becomes more pronounced as the flow rate of the plugged channel decreases. When combined with the influence of added brown noise, channels in close proximity to the plugged channels may appear to have flow rates of 40%, 60%, or 80%, or even 100%, contingent on the introduction of noise. Therefore, these challenges, combined with an imbalanced dataset, construe to a highly demanding detection and classification problem.

**Fig. 9:**
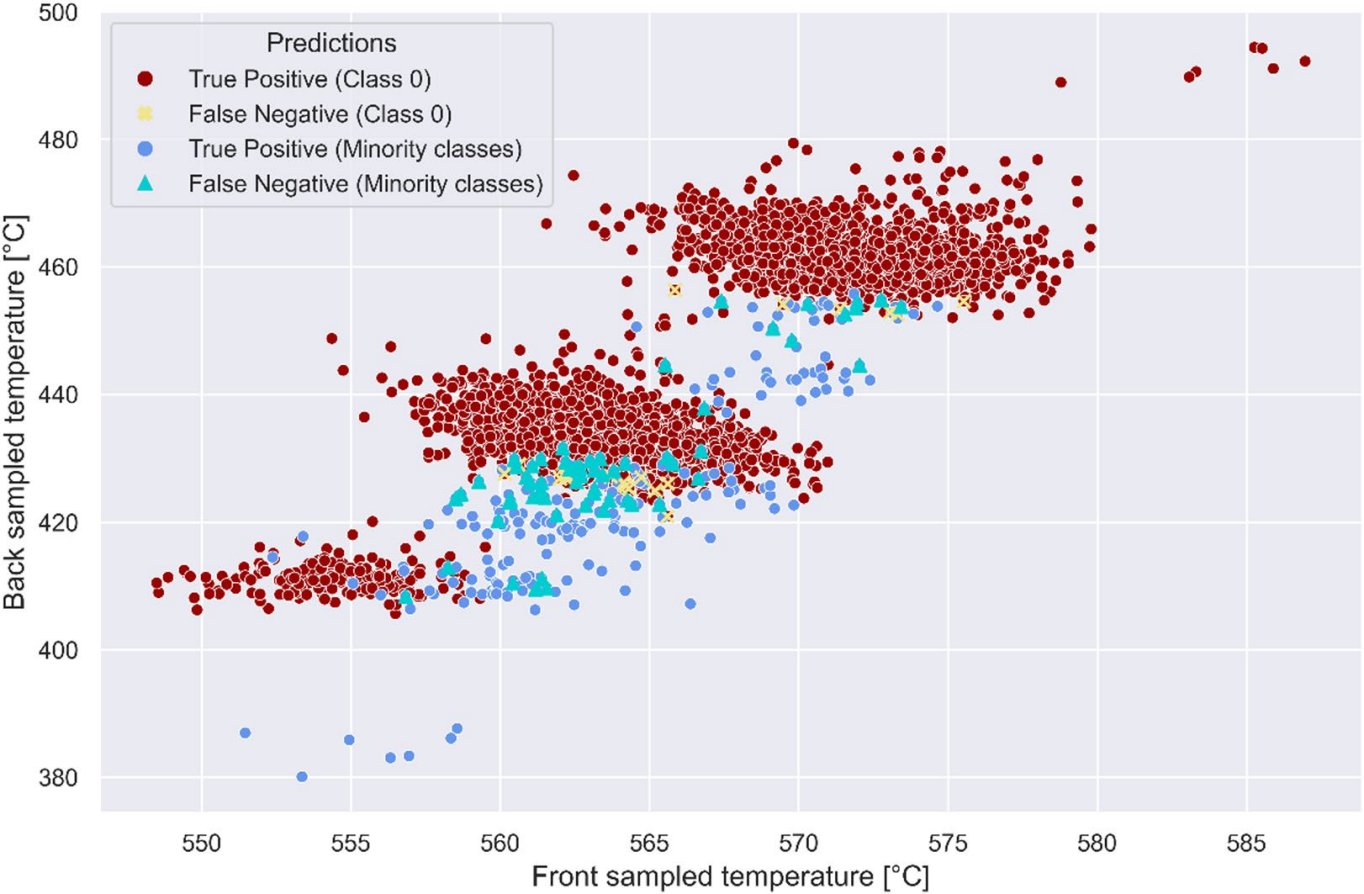
Visualization of XGBoost’s classification performance across the temperature feature space on test dataset. True positives for the majority class (class 0) are clustered in red, while true positives for minority (plugged) classes are shown in blue.

In Fig. 9, we provide a visualization of predictions made on the test dataset by the top performing classifier XGBoost. The test dataset maintains a similar minority-to-majority class ratio to the training dataset, which stand at 5.24% and 3.41%, respectively. Specifically, the test dataset consisted of a total of 90 plugged channels, with 30 instances of each of the three types of partial plugging faults as shown in Fig. 4. The synthetic DFOS gauges detect 249 instances for plugging, with class 1 (80% flow rate) accounting for 82 instances, class 2 (60% flow rate) for 84 instances, and class 3 (40% flow rate) for 83 instances. In the legend of Fig. 8, red circles represent TP for class 0 (open channels), while yellow circles indicate FN for class 0. Blue circles denote TP for the minority classes 1, 2, and 3 (plugged channels), whereas aquamarine triangles indicate FN for these same classes. The majority of FN among the minority classes belong to class 1, consistent with the results observed in Table 0. Notably, most FN for both class 0 and the minority classes occur in instances where the data distributions of two or more classes are non-separable. The highest concentration of FN is observed between class 0 and class 1, and then between class 1 and class 2, which represent the most difficult-to-detect plugging scenarios considered in this study.

**Fig. 10:**
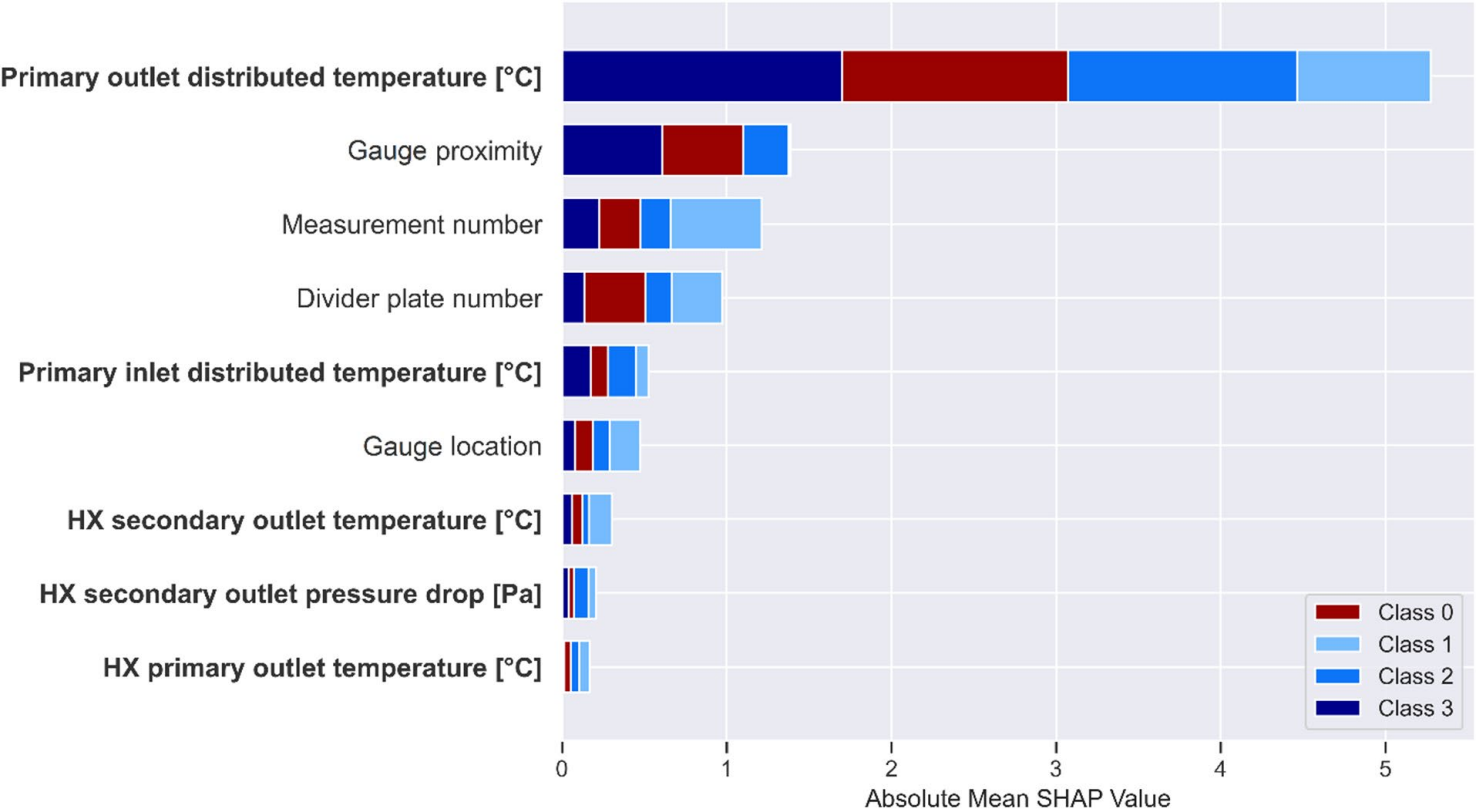
Class-specific feature importance using Shapley values.

To investigate the significance of the predictive features in the dataset, we applied SHAP (SHapley Additive exPlanations) values to XGBoost classifier output. The SHAP values are plotted in Fig. 10 to provide a visual representation of these contributions. The features having strongly influence the XGBoost model predictions are positioned higher on the vertical axis. The absolute mean SHAP value represents the average magnitude of each variable’s impact on the model’s output. The primary outlet distributed temperature emerges as the most influential variable in predicting class outcomes, exerting a significantly greater impact than all other features combined. However, while the contribution of the remaining variables is comparatively smaller, their collective effect plays a crucial role in achieving high F1-scores and AUC-PR values. Notably, relying solely on the primary outlet distributed temperature was insufficient to achieve adequate predictive performance. In fact, using only the first, fifth, and seventh variables resulted in poor model performance, underscoring the necessity of incorporating multiple contextual features to enhance classification accuracy.

**Fig. 11:**
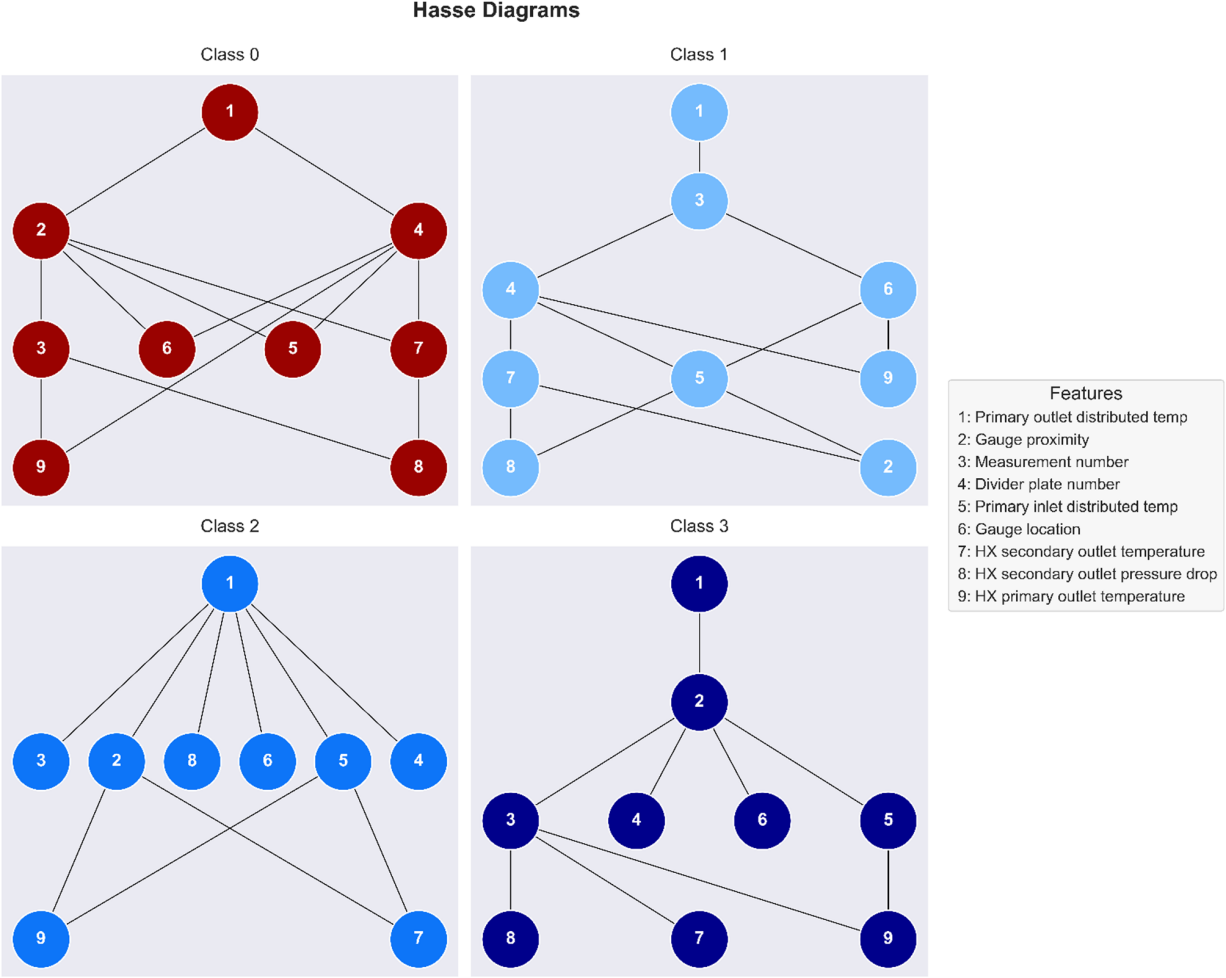
Hasse diagrams of feature importance across classes. Class-specific partially ordered sets (POSETs) constructed from Shapley values. Vertically higher nodes indicate greater feature influence. Features on the same horizontal level without connecting edges are considered incomparable, reflecting similar SHAP contributions within a class. Diagrams highlight both dominant predictors (e.g., primary outlet distributed temperature) and ambiguous feature clusters that vary with plugging severity.

To reveal class-specific differences in feature importance and explicitly represent uncertainty in their relative rankings, we constructed Partially Ordered Sets (POSETs) from SHAP values and visualized them using Hasse diagrams. Unlike traditional ranking methods that assume a strict hierarchy, POSETs allow for ambiguity by identifying features whose SHAP contributions are too similar to be reliably ordered. This is particularly important in safety-critical applications such as anomaly detection in nuclear heat exchangers, where overinterpreting marginal differences could lead to unjustified operational decisions. Figure 11 illustrates the resulting Hasse diagrams for each of the four classes. Across all classes, the primary outlet distributed temperature (Feature 1) consistently occupies the highest hierarchical level, confirming its universally dominant predictive role. However, notable class-specific variations emerge among secondary features.

For Class 0 (normal operation), the diagram reveals moderate structural complexity, with gauge proximity (Feature 2) and divider plate number (Feature 4) positioned prominently beneath the primary outlet distributed temperature, highlighting their strong secondary predictive roles under normal conditions. Secondary thermal and pressure measurements, such as HX secondary outlet temperature (Feature 7) and HX secondary outlet pressure drop (Feature 8), along with geometric indicators such as gauge location (Feature 6), form an interconnected and ambiguous cluster. This explicit representation suggests that normal operation is reliably characterized by dominant thermal gradients and localized geometric features, with other variables contributing less distinctly.

In contrast, Class 1 (20% plugging) displays a more structurally complex hierarchy. Primary outlet distributed temperature again leads clearly, followed by measurement number (Feature 3). However, the secondary level presents substantial ambiguity, grouping multiple features, divider plate number (Feature 4), primary inlet temperature (Feature 5), gauge location (Feature 6), and gauge proximity (Feature 2) into an incomparably ranked cluster. This complexity highlights that early-stage plugging events depend on subtle signals captured collectively rather than individually. Operators must therefore rely on integrating multiple feature measurements to detect mild anomalies effectively.

For Class 2 (40% plugging), the hierarchy adopts a distinctly simpler structure. Here, the primary outlet distributed temperature remains dominantly positioned, with a broad cluster of secondary features, including divider plate number (Feature 4), primary inlet distributed temperature (Feature 5), gauge proximity (Feature 2), measurement number (Feature 3), gauge location (Feature 6), and HX secondary outlet pressure drop (Feature 8), located immediately below in an explicit incomparability relationship. This simplification indicates that as plugging degree increases, the model distinctly prioritizes the primary temperature indicator, while the secondary predictive influence becomes evenly distributed across numerous supporting measurements, underscoring the need for diverse monitoring to reliably classify moderate anomalies.

Finally, for Class 3 (60% plugging), the hierarchy complexity increases once again. While the primary outlet distributed temperature remains dominant, gauge proximity (Feature 2) distinctly emerges as a critical secondary feature, sitting clearly above a cluster of comparably influential features such as primary inlet distributed temperature (Feature 5), gauge location (Feature 6), HX secondary outlet pressure drop (Feature 8), and HX primary outlet temperature (Feature 9). The structural ambiguity at lower levels emphasizes that severe plugging scenarios engage a broader and more nuanced combination of predictive cues. Consequently, accurate detection and classification of severe anomalies require a comprehensive, multi-feature measurement strategy rather than reliance on singular predictors. Overall, the class-specific POSET visualizations via Hasse diagrams provide novel interpretability insights, clearly and explicitly highlighting both dominant hierarchical relationships and crucial ambiguities among predictive features. This explicit approach enables reactor operators to better appreciate the underlying uncertainty in model predictions, enhancing decision-making robustness and operational safety.

Furthermore, the class-dependent POSET structure provides a mechanistic linkage to HX topology and transport. The Hasse diagram for Class 1 shows horizontal clusters (incomparabilities) because the incipient plug produces a localized perturbation whose diffusion length over the snapshot dwell time is comparable to the spacing of DFOS measurement points, several features thus contribute at similar, low levels. As severity increases (Classes 2–3), the partial order sharpens: features topologically closer to the primary outlet/plenum (sensor position/proximity, divider plate number) dominate more distal ones, and the primary outlet distributed temperature remains in the top strata, consistent with the thermal path from obstruction to outlet. Secondary-side pressure drop and outlet thermocouples rise in the order only in Classes 2–3, as hydraulic resistance and bulk outlet temperature respond to larger blockages. Occasional top–bottom DFOS reversals in Class 1 are consistent with weak, stratified heating under high-temperature buoyancy, while these reversals resolve as the signal grows. Sensor position and sensor proximity encode graph distance along the primary-side measurement path. We interpret POSET comparabilities with respect to this topology and the downstream thermal/hydraulic pathway to the outlets. Together, the class-specific partial orders align with expected topological constraints and diffusion/advection-driven propagation of the anomaly.

These diagrams collectively illustrate how feature importance shifts with anomaly severity. While SHAP values alone provide magnitude-based attribution, the POSET framework reveals the latent structural differences and uncertainty zones in the model’s decision logic. For example, horizontal clusters of incomparable features signal areas of interpretive ambiguity, an insight that conventional bar plots or sorted rankings obscure. Importantly, these findings are not just interpretive but operational as they guide where attention should be distributed in monitoring systems. In normal conditions, operators may rely on a few key sensors. As plugging increases, decision-making must integrate broader and more context-dependent signals. By providing both hierarchical clarity and explicit representations of ambiguity, POSET-based analysis enhances the transparency and reliability of model interpretation under varying conditions.

By combining SHAP values with the structural expressiveness of POSETs, we introduce a synergistic interpretability framework that offers more than the sum of its parts. While SHAP values quantify the individual contribution of features through a principled, game-theoretic approach, they inherently impose a total ordering, even when feature effects are indistinguishably close. The POSET formalism addresses this limitation by encoding partial rankings, thus capturing zones of uncertainty and ambiguity that are critical for trustworthy interpretation. This dual representation enables a deeper level of insight: SHAP highlights which features matter, while POSETs clarify how confidently we can distinguish among them. In practical terms, this synergy allows reactor operators to move beyond static importance scores toward a context-aware understanding of model behavior. For example, in cases of early-stage plugging, the POSET structure reveals when multiple features must be jointly considered, signaling that no single variable dominates the prediction. This approach elevates explainability from isolated rankings to a network of inter-feature relationships, directly supporting safer and more informed operational decisions. To our knowledge, this is the first application of POSETs in conjunction with SHAP for nuclear anomaly detection, providing a novel pathway for structurally grounded, class-aware interpretability in safety-critical systems.

## Methods

**Machine learning models** In this work, we detect and localize channel plugging by using classification methods. Specifically, we separate all channel plugging regardless of the plugging flow rate into four classes: open channel (class 0) and plugged channels (class 1, class 2, and class 3). Prior to the application of classifiers, it is essential to perform preprocessing of our dataset. First, we transform the categorical variables into numerical variables. Second, we perform normalization of the dataset, that is to remove the mean and scale each variable to unit variance. Furthermore, we divide the training data and testing data to 80% and 20% of the total dataset, respectively. We use 20% of the training data for validation. The testing data include cases of both single plugging flow rate and multiple plugging flow rates which result from the combination of the three distinct flow rates. In total, there are 30 channel plugging instances for each flow rate scenario. The optimization strategy necessary for the fine tuning of the hyper parameters of individual ML models was performed using the GridSearchCV module and the Stratified ShuffleSplit cross-validation technique. The utilized module takes the chosen estimator, the grid of parameters, and the cross-validation technique and performs an exhaustive search to find the best parameters. The Stratified ShuffleSplit method is a merge of StratifiedKFold and ShuffleSplit, which returns stratified randomized folds. The folds are made by preserving the percentage of samples for each class. All models were trained using the same cross-validation method to maintain consistent class distributions across folds. Table 4 summarizes the final model configurations, including both optimized and default hyperparameters used during training and prediction for each classifier.

To determine the optimal approach to detection and localization of channel plugging, we benchmark eight ML models: Logistic Regression, K-Nearest Neighbors, Gaussian Naïve Bayes, Support Vector Machines, Decision Tree Classifier, Random Forest Tree Classifier, Feed-Forward Neural Network, and Extreme Gradient Boosting Classifier.

**Logistic regression** (LR), despite its name, is primarily used for classification tasks. It is a fundamental classification model in ML that predicts the probability of an outcome based on one or more independent variables. It models the relationship between the dependent variable and independent variables by estimating probabilities using the logistic function. Logistic Regression encompasses three primary types: binary, multinomial, and ordinal. In the multi-class setting it generalizes the binary logistic (sigmoid) model via the softmax function. In this work, we employ multinomial LR to predict which of four channel-plugging classes each sample belongs to.

**K-nearest neighbors** (KNN) is a non-parametric classification algorithm. KNN predictive process involves discerning the classification of a data point by analyzing its K nearest neighbors within the feature space. KNN operates as a lazy learning algorithm, deferring the processing of training examples until predictions are required. During prediction, the algorithm identifies the K nearest neighbors of a query point, determining the class label (classification) based on their collective similarity. KNN approximates its target function locally. Unlike global approximations, local approximations offer practical advantages in learning and prediction.

**Gaussian Naïve Bayes** (GNB) is a probabilistic classification model that assumes features are distributed according to a Gaussian distribution. GNB applies Bayes’ theorem to calculate the conditional probability of a data point belonging to a particular class given its features. Despite its simplicity, Gaussian Naive Bayes is effective in domains where feature independence assumptions hold.

**Support vector machine** (SVM) is used for both classification and regression tasks. It works by finding the optimal hyperplane that separates data points into different classes, maximizing the margin between classes. Maximizing the margin distance increases confidence for correct classification of future data. SVM is effective in high-dimensional spaces and in non-liner classifications.

**Table 4:** Final hyperparameter configurations for eight ML classifiers.

Model	Descriptors
Logistic Regression	C = 6, penalty = ‘l1’, solver = ‘saga’, multi_class = ‘multinomial’, max_iter = 100
K-nearest neighbors	n_neighbors = 3, weights = ‘distance’, algorithm = ‘auto’, metric = ‘minkowski’
Gaussian Naïve Bayes	priors = None, var_smoothing = 1e-09
Support Vector Machine	C = 12, kernel = ‘rbf’, degree = 3, gamma = ‘scale’, tol = 1e-3, probability = True, decision_function_shape = ‘ovr’
Decision Tree	criterion = ‘entropy’, splitter = ‘best’, max_depth = None, min_samples_split = 10, min_samples_leaf = 4
Random Forest	n_estimators = 100, criterion = ‘entropy’, max_depth = None, min_samples_split = 2, min_samples_leaf = 1
Feed-forward Neural Network	Layer sizes = [9, 32, 16, 8, 4], activation = ‘relu’, dropout = 0.30 (after layers 2, 3, 4), optimizer = ‘sgd’, loss = ‘categorical_crossentropy’, epochs = 95, batch_size = 16, validation_split = 0.2, early stopping (patience = 50, min_delta = 0.00003)
Extreme Gradient Boosting	learning_rate = 0.05, n_estimators = 200, max_depth = 5, min_child_weight = 1, gamma = 0.4, subsample = 0.55, colsample_bytree = 0.85, objective = ‘multi: softprob’, eval_metric = ‘mlogloss’,

**Decision tree** (DT) classification model is a non-parametric algorithm that recursively partitions data into subsets based on the values of input features. DT conducts a greedy search to identify the optimal split points within a tree. It constructs a tree-like structure that can be seen as a piecewise constant approximation, and consists of the root nodes, internal nodes, and leaf nodes. Each internal node represents a feature, and each leaf node corresponds to a class label or a regression value.

**Random forest** (RF) is an ensemble learning technique that constructs multiple decision trees during training and outputs the mode of the classes for classification or the average prediction for regression. It mitigates overfitting and enhances accuracy by aggregating predictions from diverse individual trees, making it robust and suitable for various complex datasets across different domains. RF leverages both bagging and feature randomness to create an uncorrelated forest of decision trees. The integration of feature randomness generates a random subset of features, ensuring low correlation among decision trees. This distinction is pivotal: while decision trees contemplate all possible feature splits, random forests only select a subset of features, enhancing efficiency and robustness.

**Neural network**, known as multi-layer perceptron, emulate the cognitive processes of the human brain by integrating interconnected processing units akin to neurons. These units organize into layers: an input layer, representing input fields; one or more hidden layers; and an output layer, depicting target fields. The feed-forward neural network (FNN) comprises interconnected layers of neurons where information flows unidirectionally from input to output layers. It employs activation functions to introduce non-linearities, enabling complex pattern recognition. By adjusting weights and biases through backpropagation, it learns to classify data accurately. Categorical cross-entropy is often used as a loss function for multi-class classification problems.

**Extreme gradient boosting** (XGBoost) is an advanced ensemble learning technique designed for classification and regression tasks. XGBoost utilizes decision trees as base learners and employs regularization techniques to enhance model generalization. It sequentially builds a multitude of weak decision trees and optimizes them using gradient boosting algorithms. Each tree learns from its predecessors and updates the residual errors. Unlike bagging techniques such as Random Forest, XGBoost employs trees with fewer splits, enhancing computational efficiency and scalability while delivering superior predictive performance across diverse datasets and domains.

**Classification metrics** To evaluate classifier performance, we utilize various performance indicators. The selection of appropriate classification metrics is considered equally pivotal as the choice of the learning algorithm for a given classification task. In this work, we employ both threshold and ranking metrics.

Threshold metrics serve to quantify classification prediction errors, offering insights into the fraction, ratio, or rate at which predicted classes deviate from their expected class in a dataset. A disadvantage of the threshold metrics is that they assume full knowledge of the conditions under which the classifier will be deployed. Specifically, they assume that the class imbalance observed in the training set will persist consistently throughout the operational lifespan of the classifier^38^. Notably, in our dataset, this assumption aligns with the actual scenario. Specifically, here we employ precision, recall, and F1-score as classification threshold metrics, while opting to exclude the accuracy metric. The accuracy metric, while useful in datasets with balanced distributions, can be highly misleading in datasets with imbalanced distributions, as observed in our dataset. These metrics are based on the true positive (TP), true negative (TN), false positive (FP), and false negative (FN) terms. A true positive outcome occurs when the true output is correctly predicted as true.

TP represents the number channels correctly classified as plugged channels, while FP signifies the number of channels that are incorrectly classified plugged when they are open. TN denotes the number of channels correctly classified as open channels, while FN denotes the number of open channels that are incorrectly classified as plugged.

**Precision** is the ratio of true positives to the total number of true and false positives, in other words, the ratio of predicted positives to the total number of actual positives. Precision is the fraction of how many selected items are relevant. High rates of precision relate to low false-positive rates.

1 $$\:Precision=\frac{TP}{TP+FP}$$

**Recall** is the ratio of true positives to the total number of true positives and false negatives, in other words, the ratio of predicted trues to actual trues. Recall is the percentage of finding relevant cases. Thereby, accuracy and recall are all based on the relevant cases. High rates of recall relate to low rates of false negatives.

2 $$\:Recall=\frac{TP}{TP+FN}$$

**F1-score** is an evaluation metric that measures a model’s accuracy. It combines precision and recall using their harmonic mean, and maximizing the F1-score implies simultaneously maximizing both precision and recall.

3 $$\:F{1}_{score}=\frac{Precision\cdot\:Recall}{Precision+Recall}$$

**Confusion matrix** is a fundamental tool in classification tasks, offering a clear and concise summary of how well a model’s predictions align with actual data. It is a tabular representation that breaks down the classification results into different categories, primarily distinguishing between correct and incorrect predictions. The matrix is structured with actual classes in the rows and predicted classes in the columns. The diagonal elements represent true negatives and true positives, signifying the number of accurate predictions for each class. By examining the off-diagonal elements, one can assess the types of errors made by the model. For instance, elements off the diagonal in a given column denote false positives, while elements off the diagonal in a row indicate false negatives. In essence, a confusion matrix serves as a valuable tool to gauge the model’s performance, helping to identify strengths and weaknesses in the classification process with a straightforward visual representation.

Ranking metrics assess classifiers based on their ability to effectively distinguish between classes. These metrics require classifiers to predict a score or a probability of class membership. From this score, different thresholds can be applied to test the effectiveness of classifiers. Classifiers that consistently maintain high scores across a spectrum of thresholds demonstrate superior capacity for class differentiation, thereby earning higher rankings. Among the most commonly employed ranking metrics are the receiver operating characteristic (ROC) analysis and the precision-recall (PR) analysis.

ROC relies on recall and the false positive rate, calculated the ratio of total number of false positive predictions to the sum of the false positives and true negatives. It evaluates classifier performance on both classes, such as open and plugged channels. However, it can yield overly optimistic results when applied to datasets with substantial class imbalances, a scenario encountered in our study. Therefore, we have opted not to utilize this metric.

**PR** relies on precision and recall ratios. It assesses classifier performance within the minority class (plugged channels) while disregarding the majority class (open channels). It is common to investigate classifier performance using the PR curve, which is a graphical representation of recall (x-axis) versus precision (y-axis) for different probability thresholds. An adept classifier maintains high precision and recall across the curve. Although the PR curve is a valuable diagnostic tool, especially in highly skewed domains where ROC curves may be overly optimistic, it can be challenging to compare multiple classifiers based on their respective curves. Instead, we calculate the area under curve (AUC) to obtain a single score for each classifier model across all threshold values.

**AUC-PR**, a metric that summarizes the information contained within a PR curve, offers particular utility for imbalanced classification challenges like the one presented in our study, as it emphasizes the minority class. Various methods can be employed for AUC-PR calculation, including the lower trapezoid estimator, the interpolated median estimator, and the average precision. In this work we have opted for the average precision method due to its capacity for providing a more balanced assessment. In general, a higher AUC-PR score indicates enhanced classifier performance for the given task, with a perfect classifier achieving an AUC-PR value of one.

Furthermore, we can determine the baseline for AUC-PR, a pivotal aspect that delves deeper into classifier performance. The baseline value represents the performance a random or dummy classifier could achieve, and it equals the ratio of the number of samples in the positive class to the total number of samples, also known as the prevalence of the positive class. Therefore, for a comprehensive evaluation of classifier classification capabilities, comparing their AUC-PR value with the baseline is essential.

**Fig. 12:**
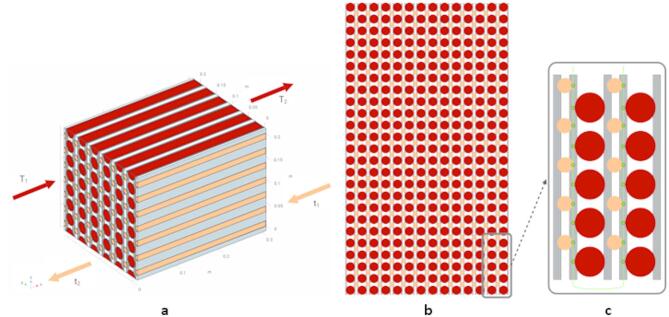
(**a**) Schematic visualization of matrix HX consisting of an array of interleaved primary (red) and secondary (orange) channels separated by divider plates. The front side of the HX is the primary fluid inlet and the secondary outlet side. The back side of the HX is the secondary fluid inlet and primary fluid outlet side. (**b**) Visualization of the front side of matrix HX. (**c**) Schematic diagram of DFOS installation along the end edges of the metallic divider plates. The red and orange circles indicate the primary and secondary tubes, respectively. The green line indicates the DFOS, where the location of gauges used in this study are marked with small green circles.

**Molten salt heat exchanger model** The primary coolant salt is NaCl-MgCl_2_ with inlet temperature of 600 °C, and the secondary coolant is solar salt KNO_3_-NaNO_3_ with fixed inlet temperature of 300 °C. Computer model of a simplified matrix HX was developed with COMSOL Multiphysics with Heat Transfer Module and Pipe Flow Module. The geometry of an internal section of the simplified matrix HX, consisting of an array of parallel primary and secondary tubes separated by metallic divider plates, is shown in Fig. 12. This figure shows only the primary inlet distributed temperature of the heat exchanger where the primary side fluid is introduced into the system. COMSOL model of the simplified matrix HX prototype consists of rows of 25 primary tubes, 25 secondary tubes, and 28 divider plates. The primary and secondary tubes have diameters of 1in and 0.5in, respectively, and wall thickness of 0.05 in. The rows of primary and secondary tubes are separated by stainless steel 316 divider plates with 0.5in thickness.

The sensors simulated with COMSOL model include a synthetic DFOS placed across the divider plates at the two ends of the HX, thermocouples placed at the combined outlets of the primary and secondary system, and pressure transmitters placed at the outlet of the second system. The synthetic DFOS consists of spatially resolved gauge segments, the readout from which can be obtained independently with an optical interrogation system. Thus, the functionality of DFOS is similar to that of a multipoint thermocouple array. However, the advantage of DFOS is that there is a single communication line with all gauges instead of multiple cables connected to each thermocouple in the array. For DTS in high temperature fluids, such as liquid salt or liquid sodium, the fiber optic sensor is inserted into a protective metallic thimble. The metallic thimble in immersed in the fluid, but the fiber silica glass is not in direct contact with the fluid, instead sensing fluid temperature through heat transfer through the thimble wall. Inserting a metallic thimble into each narrow channel would dramatically increase the complexity of heat exchanger manufacturing. Instead, in the measurement design in our study, we propose to place the metallic thimble in direct contact with metallic divider plates. In this approach, DFOS gauges measure temperature of the channels indirectly, sensing heat transfer from the channels through the divider plates. This allows for DTS without penetrating fluid carrying vessels pressure boundaries and makes the proposed sensing approach more general and less dependent on the specific heat exchanger topology. In the problem setup, synthetic DFOS readings are taken with 50 gauges from 14 divider plates (every other one in the array containing 28 divider plates). The gauges were chosen to be at locations where either the primary or secondary tubes are in contact with the divider plate. A schematic diagram of envisioned DFOS installation along the end edges of the divider plates is shown in Fig. 12. The red and orange circles indicate the primary and secondary channels, respectively. The green line indicates the DFOS, where the location of gauges used in this study are marked with small green circles.

**Fig. 13:**
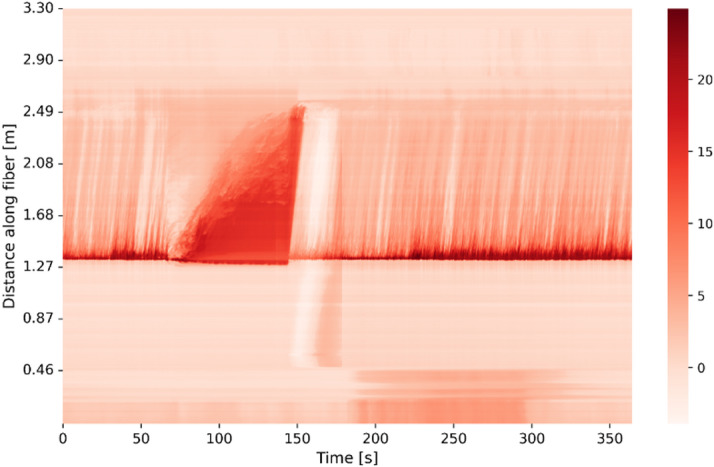
Temperature distribution in the mixing zone as a function of time and space obtained with a fiber optic sensor. Temperatures measurements are relative to a laboratory reference standard of 22.8 ºC.

**Experimental sensor noise** To model the experimental measurements with higher fidelity, in this work we introduce sensor noise into COMSOL-simulated HX data^26^. Specifically, we injected brown noise into all DTS readings, injected pink noise into the temperature readings associated with thermocouples, and injected white noise into the readings from synthetic pressure gauges. The noise spectrum for thermocouples and pressure gauges was obtained from experimental measurements with sensors installed in vessels of Mechanisms Engineering Test Loop (METL) liquid sodium experimental facility at Argonne National Laboratory. METL provides a prototypical environment of sodium fast reactor for proof-of-concept development and testing of thermal hydraulic sensors and components. The environment at METL also serves as a representative platform for molten salt reactor power plant. For the DFOS noise model, we leverage experimental measurements with Rayleigh scattering fiber optics sensors in a flow loop with water at ambient pressure and near room temperature in auxiliary facility at METL. High temperature thermal hydraulics research facilities frequently utilize experimental setups with room-temperature surrogate fluids to perform preliminary proof-of-principle studies. Water is a chemically inert fluid that has similar density and heat capacity to molten salts used in nuclear energy applications, such as FLiBe (mixture of lithium fluoride and beryllium floride). Although the measurements were obtained in water at near room temperature, the fiber optic sensor was installed in the flow loop following the same protocol as for measurements in a high temperature fluid. In addition, the photodetector noise in the interrogator device, which is located outside of harsh environment zone, would be independent of the fluid type. In the experiment, a 3.72 m-long single mode fiber optic cable without cladding was inserted into a narrow stainless steel metallic thimble. The thimble enclosing the fiber optic sensor was positioned inside a 1.5in inner diameter polycarbonate pipe of the flow loop. The Rayleigh scattering fiber measures the backscattering of light from intrinsic subwavelength defects in the fiber silica core. Local changes in fluid temperature result in local thermal expansion of the silica fiber that changes scatterers density. This backscattering signal is recorded via optical frequency domain reflectometry (OFDR), which can be converted into temperature measurements through a calibration procedure. The OFDR measurements were recorded with the Luna Optical Distributed Sensor Interrogator (ODiSI 6000). The temperature was sampled every 0.05s, with a gauge pitch of 2.6 mm along the fiber. Figure 13 shows a part of the process map image of temperature distribution in thermal mixing zone obtained with fiber optic sensor. The x-axis is the measurement time, and the y-axis is the length along the fiber. The data shown in Fig. 13, consists measurements of approximately 3.3 m long segment of the fiber for approximately 350 s duration of the experiment. The temperature readings displayed in Fig. 13 were measured relative to a laboratory reference standard of 22.8 ºC.

**Fig. 14:**
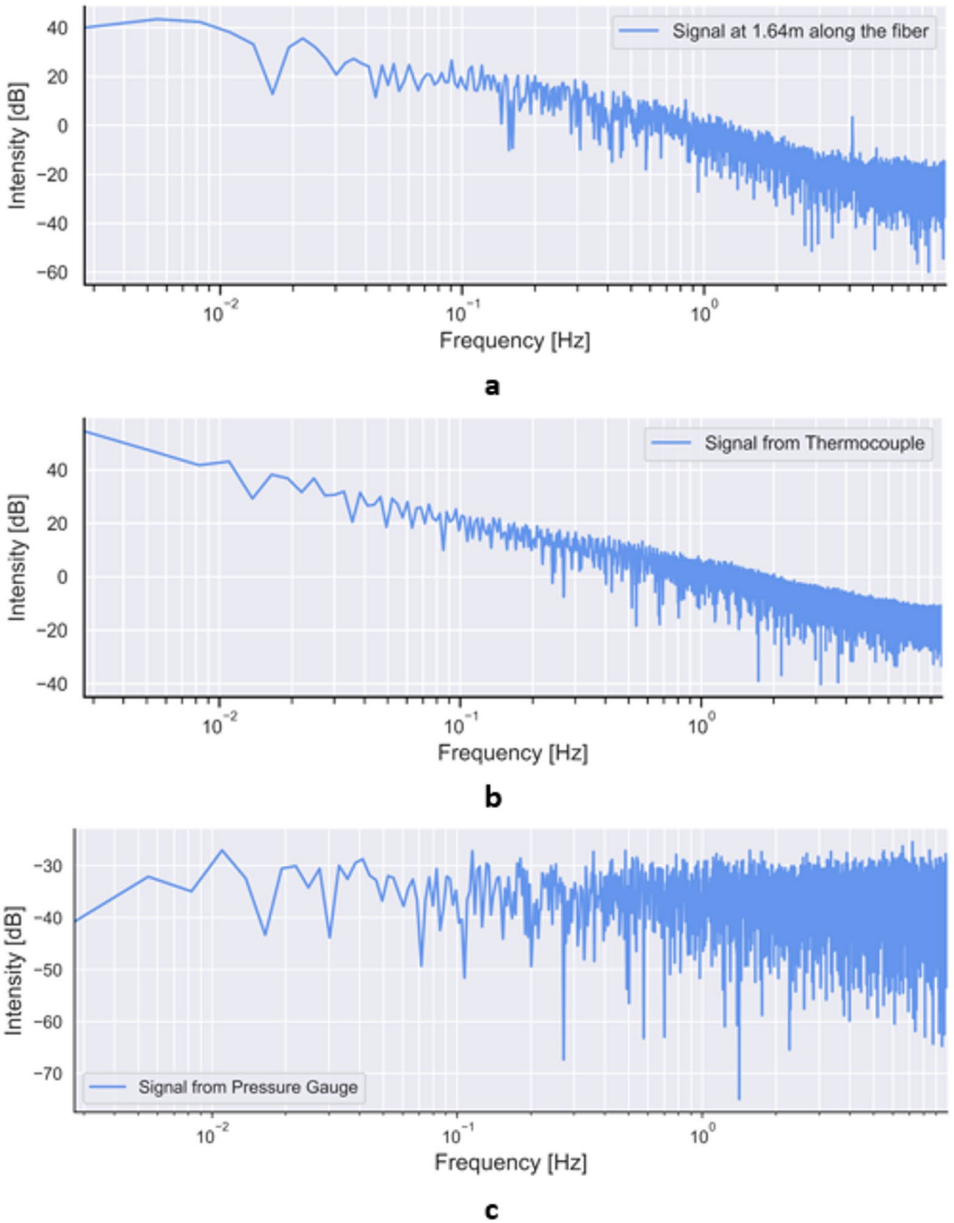
Semi log plot of frequency spectrum of the (**a**) thermal mixing zone for the gauge sensing point located at the 1.64 m mark along the fiber optic cable, (**b**) thermocouple experiment, and (**c**) pressure sensor from METL experiment.

Here, we focus on the frequency-based noise and the classification by the color of noise. First, we apply the Fourier transform to convert our continuous data into the frequency domain. Second, we calculate the power spectral density (PSD) which takes another step and calculates the power, or intensity, of the frequency content. This tool illustrates how the power or intensity of a signal varies with frequency and is easier to interpret visually than the Fourier transform. The color of the noise is determined by the shape of the power spectrum. Noise is classified by the spectral density, which is proportional to the reciprocal of frequency f raised to the power of beta. The characterization relation is given as:

4 $$\:PSD\propto\:\frac{1}{{f}^{\beta\:}}$$

where $\:\beta\:\ge\:0\:$. Each beta value corresponds to a different color of noise. When beta equals zero, noise is described as white, when beta equals one, noise is described as pink, and when beta equals two, noise is described as brown. To investigate the noise characteristics in the temperature time-series recorded by fiber optic sensors, we analyzed the PSD of the signals. Figure 14 shows the frequency spectrum of the gauge sensing point located at the 1.64 m position along the fiber optic cable. This gauge is chosen because this sensing point is exposed to significant fluctuations in the temperature transient. The x-axis denotes the frequency in a logarithmic scale, measured in Hertz (Hz), while the y-axis represents the amplitude of the frequencies in the signal, measured in decibels (dB). Figure 14 demonstrates that the spectrum exhibits a negative slope, indicating a decrease in power as the frequency increases. The decrement in power, measured in dB per decade, is consistent with the other signals and amounts to 20dB. Based on this rate of decrease, the noise in the signal corresponds to brown noise.

**Fig. 15:**
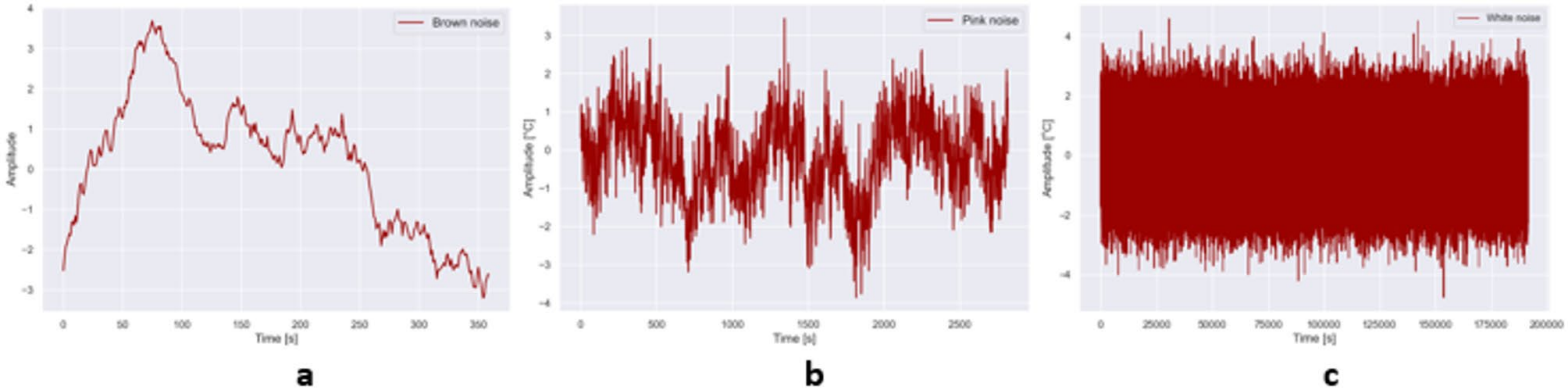
Sample of generated colored noises. (**a**) Brown noise related to fiber optic sensors’ experiment that ran for 370 s. (**b**) Pink noise related to thermocouple sensors’ experiment that ran for 2820 s. (**c**) White noise related to pressure sensors’ experiment that ran for 191,754 s.

Following the classification of colored noise in the experimental data, Fig. 15 displays a sample of the brown noise generated for injection into the synthetic temperature dataset of HX model obtained with COMSOL computer simulations. Note that COMSOL produces steady-state values, while in the experiment the operator observes a heat map similar to that in Fig. 13. The addition of noise changes the synthetic dataset, bringing it closer to experimental. This process aids in creating datasets that more realistically represent experimental conditions, which helps with testing ML algorithm and reducing uncertainty in their performance on purely experimental data.

Furthermore, we show that the classifier performance does not depend on a specific noise spectrum. To examine whether our conclusions depend on a specific molten-salt noise spectrum, we subjected the unchanged, originally trained XGBoost model to three stress tests that alter spectral shape (Tests A and B) and transient sparsity (Test C) while preserving per-case DFOS variance (constant signal-to-noise ratio) and constraining DFOS deviations to a realistic 1–2 °C median absolute change. Therefore, any performance changes cannot be attributed to amplitude inflation/deflation but to the intended PSD/transient differences.

Specifically, Tests A and B steepened the test dataset’s spectrum toward $\:\beta\:=1.5$ and $\:\beta\:=2.5$, respectively. The colored-noise slope was flattened using a band-limited phase preserving Fast Fourier Transform (FFT) reweighting of the residual magnitude spectrum (mid-band). In Test C, we injected sparse short-width spikes plus a small brown background ($\:\beta\:=2$). In every test, thermocouples and cold-side pressure received only small per-case offsets to mimic metrology bias, without changing their variance.

To isolate spectral content from amplitude effects, we modified the DFOS fields $\:{x}_{c,k}\left[n\right]$ (case $\:c$; channel $\:k\in\:\left\{top,\:bottom\right\}$) while preserving the within-case variance. In our dataset, a case c is one steady operating condition / plug level (e.g., 0%, 20%, 40%, 80%), and a DFOS channel $\:k\in\:\left\{top,\:bottom\right\}$ is the temperature profile measured along the exchanger. For a given case $\:c$ and channel $\:k$, the DFOS sequence $\:{x}_{c,k}\left[n\right]$ (indexed by sensor position $\:n$) has a mean trend $\:{\mu\:}_{c,k}$​ and a residual $\:{r}_{c,k}\left[n\right]$ whose within-case variance $\:{\sigma\:}_{c,k}^{2}=var\left({r}_{c,k}\right)$ captures the amplitude of fluctuations within that case only (i.e., across sensor positions for the same operating condition). For each case we subtracted the per-case mean $\:{\mu\:}_{c,k}$ to obtain residuals $\:{r}_{c,k}\left[n\right]={x}_{c,k}\left[n\right]-{\mu\:}_{c,k}$ with $\:{\sigma\:}_{c,k}=std\left({r}_{c,k}\right)$. After every manipulation, we renormalized to $\:{\sigma\:}_{c,k}$ so the signal-to-noise ratio (SNR) per case is unchanged.

When probing sensitivity to spectral content (autocorrelation, β-slope) or transient sparsity (spikes), it is essential to hold $\:{{\upsigma\:}}_{\mathrm{c},\mathrm{k}}$​ fixed for every case and channel. First, it keeps the SNR constant within each class, so any change in performance cannot be attributed to simple amplitude inflation or deflation. Second, it isolates the effect of how energy is distributed over frequencies (PSD shape) and time-localization (spikes) from the confounding effect of how much energy is present. Third, it avoids artificial class-overlap changes that would occur if one class’s variance increased while another’s did not, which would bias confusion matrices and AUC-PR. Fourth, it provides a conservative test of robustness as the classifier is not helped or hurt by trivial SNR changes because only spectral/temporal structure is perturbed. In practice we therefore renormalize every manipulated residual back to $\:{\sigma\:}_{c,k}$ (std-ratio ≈ 1.00 per case), ensuring that observed performance shifts are genuinely due to spectrum or spikes, not SNR.

For Tests A and B, we modified the magnitude spectrum of $\:{r}_{c,k}$​ in a mid-band $\:\left[{f}_{lo},{f}_{hi}\right]$ to emulate colored-noise slopes $\:1/{f}^{\beta\:}$ while leaving phases intact. We estimate the baseline colored-noise slope $\:\widehat{\beta\:}$ from the Welsch PSD by a mid-band log-log fit $\:{\mathrm{log}}_{10}PSD\left(f\right)=\alpha\:\cdot\:{\mathrm{log}}_{10}f+\beta\:$. To impose a target slope $\:{\beta\:}_{target}\in\:\left\{1.5,\:\:2.5\right\}$ we reweight the FFT magnitudes of $\:{r}_{c,k}$ on the midband $\:f\in\:\left[0.01,\:0.45\right]$ cycles/sample using a blender multiplier: $\:{w}_{blend}\left(f\right)=\left(1-\alpha\:\right)+\alpha\:\cdot\:{f}^{-\varDelta\:\beta\:/2}$ and $\:\varDelta\:\beta\:={\beta\:}_{target}-{\widehat{\beta\:}}_{baseline}$. Here, $\:\alpha\:$ is the frequency-domain blend ($\:\alpha\:\in\:\left[\mathrm{0,1}\right]$, where zero means no tilt and one means full tilt) and $\:\widehat{\beta\:}$​ is the mid-band log–log slope of the Welch PSD. We applied $\:{w}_{blend}$​ only for $\:f\in\:\left[0.01,\:0.45\right]$ to avoid DC/Nyquist artifacts), formed $\:\stackrel{\sim}{R}={w}_{blend}\cdot\:R$, inverse transformed to $\:\stackrel{\sim}{r}$ and did a light time-domain blend to limit pointwise drift: $\:{r}^{*}=\left(1-\eta\:\right)r+\eta\:\cdot\:norm\left(\stackrel{\sim}{r}\right)$, with $\:std\left({r}^{*}\right)=\sigma\:\left(per\:case\right)$, where $\:\eta\:$ is time-domain blend between the original residual and the tilted residual. We then set $\:{x}_{c,k}^{{\prime\:}}={\mu\:}_{c,k}+{r}_{c,k}^{*}$. To make the perturbation non-negligible yet realistic, we capped the median absolute pointwise change to $\:med\left(\left|{x}^{{\prime\:}}-x\right|\right)\approx\:1-2~^{\circ}{\rm C}$ across DFOS samples and renormalized per case if needed. For Test A we used a band 0.01–0.45 cycles/sample, for the front side of HX DFOS temperature (top) $\:\alpha\:\approx\:0.55$, $\:\eta\:\approx\:0.50$ and for the back side of HX DFOS temperature (bottom) $\:\alpha\:\approx\:0.60$, $\:\eta\:\approx\:0.55$, while achieved a median $\:\left|\varDelta\:\right|$: $\:top\approx\:1.7\:~^{\circ}{\rm C}$ and $\:bottom\approx\:1.0\:~^{\circ}{\rm C}$. For Test B we used the same band, for both top and bottom $\:\alpha\:\approx\:0.35$ and $\:\eta\:\approx\:0.30$, while achieved a median $\:\left|\varDelta\:\right|$: $\:top\approx\:1.3\:~^{\circ}{\rm C}$ and $\:bottom\approx\:2.0\:~^{\circ}{\rm C}$. The $\:\beta\:=1.5$ flattening required slightly stronger reweighting (higher $\:\alpha\:$) and replacement (higher $\:\eta\:$) to realize a measurable slope change while staying within the 1–2 °C cap, while the $\:\beta\:=2.5$ steepening is obtained with moderate settings.

For Test C, we added sparse bursts plus a small brown component to each DFOS residual: $\:{r}^{new}=r+s+z$, where $\:s$ is a short-width spike train ($\:rate\approx\:1-1.2\%$ of positions, width 1-3 samples, and Gaussian amplitudes with $\:3\sigma\:\approx\:peak$), and $\:z$ is a small $\:\beta\:=2$ component. After mean removal, variance renormalization, and a light blend $\:{r}^{*}=\left(1-\eta\:\right)r+\eta\:\cdot\:norm\left({r}^{new}\right)$ with $\:\eta\:\approx\:0.35$, we capped $\:med\left(\left|{x}^{{\prime\:}}-x\right|\right)\approx\:1-2~^{\circ}{\rm C}$ and re-normalized per case. Achieved median $\:\left|\varDelta\:\right|$: $\:top\approx\:1.6~^{\circ}{\rm C}$, $\:bottom\approx\:1.9~^{\circ}{\rm C}$.

**Fig. 16:**
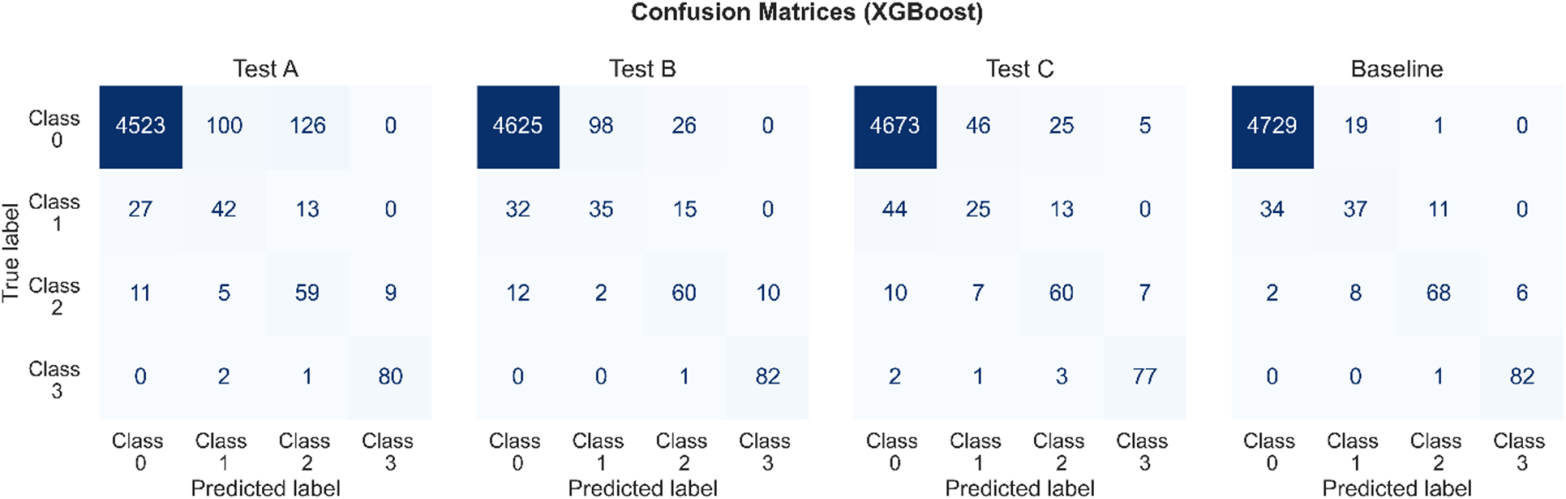
*Confusion matrices (XGBoost*,* Tests A – C and baseline)*: Variance-preserving spectral tilts to $\:\beta\:=1.5$ (Test A) and $\:\beta\:=2.5$ (Test B), and variance-preserving mixed spikes (Test C), leave classwise counts close to baseline. Class 1 remains the dominant error mode.

Figure 16 shows that confusion matrices remain strongly diagonal-dominant across all stress tests and the baseline. Class 0 maintains very high recall (correct counts 4523, 4625, 4673 in Tests A–C; 4729 in baseline), indicating that departures from the baseline PSD do not spuriously inflate false alarms. Class 3 (severe plugging) also remains high-recall (80–82 correct in A/B; 77 in C; 82 in baseline), with only a small degradation under mixed spikes. Class 2 is stable (≈ 59–60 correct across conditions, ~ 71% recall). The incipient class (Class 1) remains the most challenging boundary case, with recall declining from ~ 51% (42/82) in Test A to ~ 43% (35/82) in Test B and ~ 31% (25/82) in Test C; its errors split primarily toward Classes 0 and 2, consistent with modest overlap induced by autocorrelation/transient changes at constant SNR. Overall, the class ordering and dominant error mode are unchanged by spectral tilts or spikes.

**Fig. 17:**
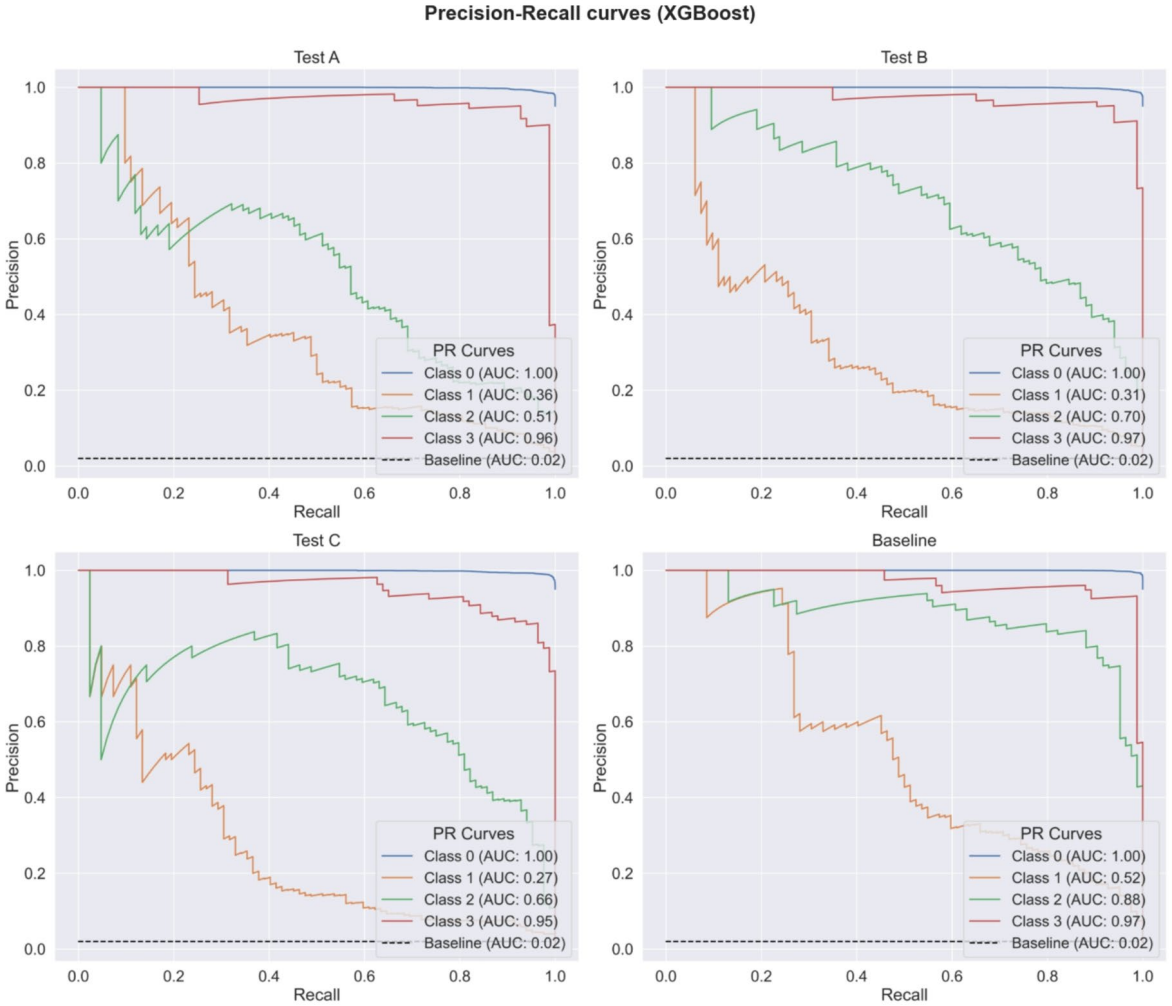
*Precision-Recall curves and Area Under the Curve values (XGBoost*,* Tests A – C and baseline)*: At constant SNR, Classes 0 and 3 remain near ceiling. Class 2 is stable/slightly improved under PSD tilts and mixed spikes. Class 1 shows a modest decrease (AUC ≈ 0.27–0.31), indicating limited sensitivity to spectral shape and transient sparsity rather than amplitude.

Figure 17 shows that AUC–PR curves change only slightly relative to baseline. Classes 0 and 3 remain near ceiling (AUC ≈ 1.00 and 0.95–0.97). Class 2 is stable or slightly improved under spectral tilts and mixed spikes (AUC ≈ 0.66–0.70), whereas Class 1 exhibits a modest decrease (AUC ≈ 0.27–0.31). Because per-case variance is matched exactly, these shifts cannot be attributed to SNR. Instead, they reflect a genuine but limited sensitivity to autocorrelation and transient structure. The overall ranking and trends are unchanged, indicating that our conclusions are insensitive to reasonable departures from an exact molten-salt PSD.

Other PSD-altering perturbations are possible. Our aim here was to test representative departures along two orthogonal and commonly observed axes in instrumentation noise: (i) the stationary spectral slope of colored noise (β-tilts spanning $\:\beta\:=1.5-2.5$, i.e., from pinkish to dark-brown regimes), and (ii) transient sparsity via mixed spikes at constant SNR. These change autocorrelation/PSD shape and time-local bursts without confounding effects from amplitude (per-case variance is preserved) or model adaptation (the classifier is not retrained). The fact that XGBoost’s AUC-PR and confusion matrices shift only modestly under both stationary (Tests A–B) and transient (Test C) perturbations provides convergent evidence that our conclusions are not contingent on a specific PSD.

**Shapley values** Shapley values offer a principled approach to quantify the influence of individual features on model predictions. Rooted in cooperative game theory, these values provide a consistent and fair attribution of prediction contributions across features, ensuring that each feature’s impact is accurately represented irrespective of model complexity. Key advantages include their model-agnostic applicability, ensuring versatility across various algorithms, precise local explanations, which illuminate the decision-making process for individual predictions, and the adherence to properties such as additivity and consistency, which guarantee coherent and reliable interpretations. The SHAP^39^ (Shapley Additive exPlanations) explainability framework harnesses these principles, offering a robust toolkit for elucidating the inner workings of ML models. By leveraging SHAP, this work presents the contribution of each input variable towards the prediction of the output so deeper insights into the models can be gained, fostering transparency and trust in AI systems.

To analyze overall feature importance, we aggregated the absolute Shapley values across all samples and generated bar plots ranking features by their mean absolute contribution. For each prediction, the Shapley value $\:{\phi\:}_{i}$ of feature $\:{f}_{i}$ represents the average marginal contribution of that feature across all possible coalitions of features, enabling precise, locally faithful explanations. These plots were used to identify consistently influential features and to inform subsequent construction of Partially Ordered Sets (POSETs) for deeper structural analysis. By incorporating Shapley values into our interpretability framework, we gain both numerical quantification of feature impact and a transparent foundation for downstream interpretability tools such as POSET-based Hasse diagrams.

**Partial order sets** Partial order sets (POSETs) provide a mathematically rigorous framework for expressing and analyzing hierarchical relationships between elements that are not necessarily comparable in a total order. A POSET is defined as a set $\:P$ equipped with a binary relation $\:\le\:$ that is reflexive, antisymmetric, and transitive: for any $\:a,b,c\in\:P$, it holds that $\:a\le\:a$ (reflexitivity), $\:a\le\:b$ and $\:b\le\:a$ imply $\:a=b$ (antisymmetry), and $\:a\le\:b$, $\:b\le\:c$ imply $\:a\le\:c$ (transitivity). In this study, POSETs were constructed from SHAP values calculated for each class of the XGBoost classifier. Specifically, for each class, we defined a partial order over the feature set by comparing the mean absolute SHAP values and declaring $\:{f}_{i}\le\:{f}_{j}$ if the contribution of feature $\:{f}_{i}$ was at least 10% less than that of $\:{f}_{j}$, i.e., if $\:\left|{\phi\:}_{i}\right|\le\:0.9 \cdot \:\left|{\phi\:}_{j}\right|,$ where $\:{\phi\:}_{i}$ and $\:{{\upphi\:}}_{j}$ are the mean SHAP values for features $\:{f}_{i}$ and $\:{f}_{j}$, respectively. This thresholding approach ensures that only statistically meaningful differences in feature contributions are considered significant, preventing overinterpretation of marginal differences and reducing noise-induced artifacts in the order structure.

To visualize the resulting POSETs, we generated Hasse diagrams for each class, which graphically represent the minimal covering relations without redundancy from transitivity. These diagrams provide an intuitive graph-based representation of the POSET, highlighting not only which features are consistently influential but also which features exhibit ambiguous or class-specific roles. In these diagrams, nodes represent features, and edges denote strict dominance relationships. Features placed on the same horizontal level are incomparable, their SHAP values differ by less than the 10% threshold, and thus no direct order is enforced. This incomparability is a key strength of the POSET formalism—it allows for meaningful modeling of structural ambiguity that traditional total-ranking methods cannot express. By acknowledging that some features may have similar, indistinguishable influence, the POSET framework avoids artificial hierarchies and reflects the uncertainty present in real-world data. This visualization facilitates the inspection of dominance relations and the identification of feature clusters with similar predictive strength. These POSETs are constructed independently for each class, allowing the interpretability framework to adapt to the class-specific structure of feature importance.

Beyond ranking features, POSETs are broadly useful in ML contexts where elements are partially distinguishable, such as comparing model variants, evaluating hyperparameters, or understanding group behavior under non-uniform conditions. In this work, their integration into the SHAP-based interpretability pipeline offers a principled method to expose dominance, equivalence, and uncertainty within class-specific explanations, enhancing both clarity and operational relevance in safety-critical systems like nuclear heat exchanger monitoring.

Table 5 presents an end-to-end methodological workflow summarizing the data, modeling procedures, and resulting outputs.

**Table 5:** End-to-end methodological flow.

Step #	Procedure
1 (Inputs)	DFOS temperature fields (top/bottom channels). Conventional sensors (HX primary/secondary outlet TCs, secondary-side pressure drop). Contextual features (sensor position, sensor proximity, measurement number, divider plate number).
2 (Pre-processing)	Collect fewer DFOS sample points, as shown in Fig. 12. Standardize continuous features only on the training set and then apply to validation/test and deployment data (prevents leakage).
3 (Grouped data split)	Train/validation/test grouped by case (no case appears in more than one split; prevents leakage from the case index).
4 (Classifier training)	ML classifiers trained on the training set and hyperparameter tuning.
5 (Predictions & Primary evaluation)	Predict on held-out dataset. Evaluate using test metrics per class (confusion matrices, precision/recall/F1-score, PR curves, PR-AUC value).
6 (Interpretability)	SHAP values computed per class. POSET construction per class and Hasse diagrams.
7 (Deterministic localization)	Channel mapping using contextual features (sensor position, divider plate number, sensor proximity) to output the exact channel index.
8 (Deployment path)	Real-time prediction is sub-millisecond per snapshot on CPU. Outputs: severity class + channel index + explanations (SHAP/POSET).

## Data Availability

The datasets used and/or analyzed during the current study are available from the corresponding author on reasonable request.

## Code availability

The codes used and/or analyzed during the current study are available from the corresponding author on reasonable request.
